# Beauty growth-mindset promotes prosocial and altruistic behavior

**DOI:** 10.1038/s41598-024-82134-y

**Published:** 2024-12-04

**Authors:** Iris W. Hung, Natalie T. Faust

**Affiliations:** 1 Department of Marketing, The Chinese University of Hong Kong, Shenzhen, China; 2 Department of Marketing, Nova School of Business and Economics, Carcavelos, Portugal

**Keywords:** Psychology, Human behaviour

## Abstract

The effects of beauty on judgment and behavior are well-established and somewhat “unidirectional” (i.e., it seems that only beautiful people reap social benefits). In particular, a person’s beauty does not seem to have any effect on his/her own prosocial behavior. In the current research, we focus on how people relate themselves to beauty, namely beauty mindset, and how it may shape prosocial and altruistic behavior. We present 10 experiments (*N* = 4,449). Participants who hold a beauty growth-mindset (i.e., believing that beauty is improvable) donated more money to charity (vs. fixed-mindset), were more likely to get vaccinated against COVID-19 after exposure to an intervention focusing on herd-protection (vs. self-protection), and showed increased intention to engage in other forms of prosocial and altruistic behavior. Empirically, these effects are mediated by a self-perception of the ability to exert impact on others. The present research serves as a starting point for investigating how beauty growth-mindset brings profound societal effects such as promoting prosociality.

**Supplementary Information:**

The online version contains supplementary material available at 10.1038/s41598-024-82134-y.

## Introduction

Global challenges including poverty, pandemics, climate change, and a long list of people waiting for organ transplants remain in the world. Fortunately, it is generally believed that these challenges can be lessened if more people and institutions are altruistic to one another more often. For example, altruist non-profit organizations have helped to reduce malnutrition and malaria and increase access to health care for people in low-income countries^1^. Previous research has also provided evidence that altruism can contribute to solve these challenges, such that altruistic individuals are more likely to engage in pro-social behaviors to protect others during the pandemic^2^ and altruism has also been shown to increase climate policy support and pro-environmental political preferences^3^. This explains in part why researchers in economics, health care, psychology, and management have been interested in understanding the motives underlying prosocial behavior^4–10^. Approaches including cognitive and developmental, personality, social norms and group influences, personal values, affect and arousal have identified factors that produce positive effects on altruism^11–19^. Our studies show that apart from these factors, people’s mindset about beauty, which is a fundamental human attribute, can bring profound societal effects on prosociality and altruism. We define prosociality as behavior that benefits others and altruism as behavior that benefits others but is costly to the actor^11,20^. These behaviors include sharing, donating, and helping. Specifically, people who hold a beauty growth-mindset (vs. fixed-mindset), a belief that beauty is improvable with effort, are more likely to engage in prosocial and altruistic behavior (we use these terms interchangeably in this research). This heightened effect of beauty growth-mindset on prosocial behavior can be explained by two aspects of beauty growth-mindset we conjectured: beauty growth-mindset is negatively related to a focus on how one is evaluated by others^21^ and is positively related to a belief that one can make a positive impact on others^22^. We provide evidence for the latter.

## Beauty elicits social aesthetic pleasure

Beauty has interested laypersons, philosophers, and scientists for centuries. Indeed, beautification is an essential everyday ritual for many people. The global beauty products market is expected to be $736.79 billion in 2028^23^. Humans use beauty, just like other important attributes such as personality, morality, and intelligence, to understand and evaluate themselves and others. Previous research examining the link between beauty and altruism mostly focus on how attractive people are often treated altruistically^24–29^. In the current research, our departure is a focus on the relationship between a certain conceptualization of beauty and prosocial behavior. Specifically, we examined how beauty mindsets may shape altruism.

Attractive children and adults are perceived to be more intelligent and socially competent than unattractive ones^24–28^, and are offered more help and charitable giving compared to unattractive individuals^27,29^. Nevertheless, findings supporting the idea that “beautiful people are altruistic” have been sparse, limited, and mixed—only few studies with small sample sizes have been conducted, comparisons were made between attractive and unattractive conditions (thus one cannot tell the direction of effects), mixed findings were yielded, and studies almost entirely focused on how attractive people are perceived and treated by others (e.g., attractive people are often treated altruistically). Beauty has no effect on perceptions of characters such as kindness^30^. In addition, beautiful people do not seem to perceive themselves to be more altruistic and more likely to engage in prosocial behavior than unattractive people^27,30,31^. Further, meta-analyses conducted identified no effects of physical attractiveness on perceptions of kindness and concern for others^27,30,31^.

Focusing on how people relate to beauty through beauty mindsets, we assume that beauty is a form of positive social impact. Essentially, beauty automatically shares aesthetic pleasure to the external environment intrinsically without being externally justified^32,33^. Regardless of whether a beauty provider is appraised or treated positively^34,35^, an audience automatically derives aesthetic pleasure from the beauty provider. The elicitation of aesthetic emotions is automatic without much conscious attention and thus it is hard for beauty audience to ignore^36^. Beauty also possesses reward values; beautiful faces activate the reward circuitry in our brains^37^. Even infants enjoy looking at beautiful faces more than non-beautiful ones^38^. Furthermore, beauty has a strong social dimension. It is immediately visible compared to other human attributes such as intelligence and morality. As philosopher Sartwell reasoned, beauty is a pleasurable experience people naturally want to *share*^39,40^. It is *both* in the object and in the eyes of the beholders.

## The drastic influence of mindsets on human behavior

It is well-established that to understand how human attributes such as intelligence, personality, and beauty may promote positive behavior, one may do so by understanding how implicit theories (also called mindsets in social psychology; they are used interchangeably in the present research) shape people’s responses and behavior. Research has shown that the mindsets people have about human attributes produce a strong impact on behavior and decision making^41–48^. Numerous studies have demonstrated in both lab and field that intelligence mindsets have strong influence on academic achievement^49–52^. People who hold a growth mindset of intelligence believe that intellectual ability can be developed, whereas people who hold a fixed mindset believe that their intellectual abilities are immutable and fixed. In a field study conducted in the United States, over 7,000 first-year students admitted to a high-quality public university were randomly assigned to conditions with different mindset interventions (vs. no intervention in the control condition). One of these mindset interventions was a growth-mindset intervention. The intervention material was embedded in the pre-orientation folder. Based on the design and the standardized instrument employed in past research, the growth-mindset intervention used an article about research findings in neuroscience which conveys that intellectual ability is improvable with ongoing effort expended on challenging tasks. Results showed that the growth-mindset intervention significantly raised the first-year full-time course enrollment (i.e., completion of suggested credit requirement of the first year), which is a primary indicator of on-time graduation, and first-year GPAs of disadvantaged students, thus significantly reducing the socioeconomic achievement gap between disadvantaged and advantaged students^49,53–57^. In a national survey in Chile, mindsets were measured among all 10th grade public school students. Data showed that students who hold a growth (vs. fixed) mindset had higher scores in the national standardized tests in mathematics and language skills across different socioeconomic groups. In addition, students who come from low-income families are twice as likely to hold a fixed mindset than those from higher income families^58^. These studies suggest that (1) the instrument for activating a growth mindset is simple and reliable in both lab and field and (2) effects of mindsets are deep-seated.

Past research has examined the underlying mechanism of the effects of implicit theories of intelligence on behavior. Implicit beliefs are mindsets that people use to encode external information, construct imagined experiences, and form social and self-related judgments^59,60^. When it comes to academic pursuit, students who hold a growth mindset of intelligence are inherently engaging in acquisition of knowledge and are therefore more concerned about learning and mastering their skillsets whereas students who hold a fixed mindset are more concerned about performance itself. As a result, when students encounter setbacks or negative feedback, students with a fixed mindset tend to give up more easily in order not to “perform” badly (i.e., avoid signaling poor performance to the self and others). Those with a growth mindset tend to embrace setbacks as challenges. They tend to be intrinsically motivated in their pursuit, and consequently, are more likely to gain mastery in academic pursuit than do those with a fixed mindset^52^. This has an implication for our research on the link between beauty mindsets and prosociality because if such a link exists, these mindsets may similarly have a drastic impact on this outcome behavior we considered.

## Effects of beauty growth-mindset on self-perception and behavior

Research has provided robust evidence supporting the domain specificity of mindsets. Thus, it is plausible that one may hold a growth mindset in intelligence and a fixed mindset on other human attributes such as personality. Few research has focused on beauty mindsets^21,61^. Findings from these studies on beauty mindsets provide a strong foundation on which we develop the present conceptualization. First, people do hold a beauty mindset. Second, people with beauty growth- and fixed-mindsets *differ* dramatically on how they relate themselves to both the pursuit of beauty and the external world. A recent study showed that people who hold a beauty fixed mindset feel a heightened social pressure to change their physical appearance compared to those who hold a beauty growth mindset^21^. Given these findings, we conjecture that people who believe that beauty is improvable with effort may inherently engage in improving beauty whereas those who believe that beauty is fixed care about how they are evaluated by others. Furthermore, we predict that these two beauty mindsets may *relate* people to the external world *differently*. Past research showed that people appreciate beauty automatically^36,62^. It implies that people who inherently engage in improving beauty automatically *share* aesthetic pleasure with others. This (automatic) act of sharing something positive with others may shape people’s belief about the self, based on self-perception theory^22^. We conjecture that people who intrinsically pursue beauty would form a belief that they can make a positive impact. Indeed, our studies showed that among participants who are primed with beauty growth- (vs. fixed-) mindset, in a subsequent, unrelated situation where people are given an opportunity to engage in prosocial behavior, they are more likely to do so. We conjecture that people who hold a beauty fixed mindset may be less likely to hold such a perception, presumably because they focus their attention on how they are evaluated by others.

Although the present set of studies we reported are lab and online studies, we believe that the experimental results may generalize to the field for several reasons. First, we developed our beauty mindset instruments based on the standardized instrument used in well-established research on growth mindset. As noted earlier, extensive research conducted in lab or large-scale field studies have demonstrated the robustness of the effects of growth mindset of intelligence on academic achievement. In these lab and field studies, the instrument for activating growth mindset vs. fixed mindset used were largely the same. This suggests that as a starting point we should develop and test our instrument for activating beauty mindsets before we test its generalizability in the field. Second, we considered real altruistic behavior. Study 1 asked participants to consider making an online donation to a project on GoFundMe, a platform that solicits online charitable donations from the crowd. In Study 2, participants were told that we would make a real donation on behalf of them. They were then asked to allocate monetary donations to a more vs. less empowering charity project. Third, we considered various forms of altruistic behavior in different real-life contexts. Studies 3 to 6 tested the relationship between getting COVID-19 vaccination and beauty mindsets. Studies 7 to 10 concerned the intention for supporting important causes through voting, signing a petition to fight against domestic violence, and supporting a stigmatized identity.

We conducted 10 studies to systematically test our predictions on the effects of beauty mindsets on altruism. In all studies, growth (vs. fixed) mindset was either experimentally varied or measured (i.e., chronic tendency was measured). After the activation or measure of beauty mindsets, participants were introduced an altruistic giving opportunity. Studies 1 to 10 investigated effects of beauty growth-mindset on various forms of altruistic behavior. Studies 1, 4, and 9 were pre-registered (please refer to SI S1 for deviations from preregistration in Studies 1 and 4).

## Results

**Fig. 1:**
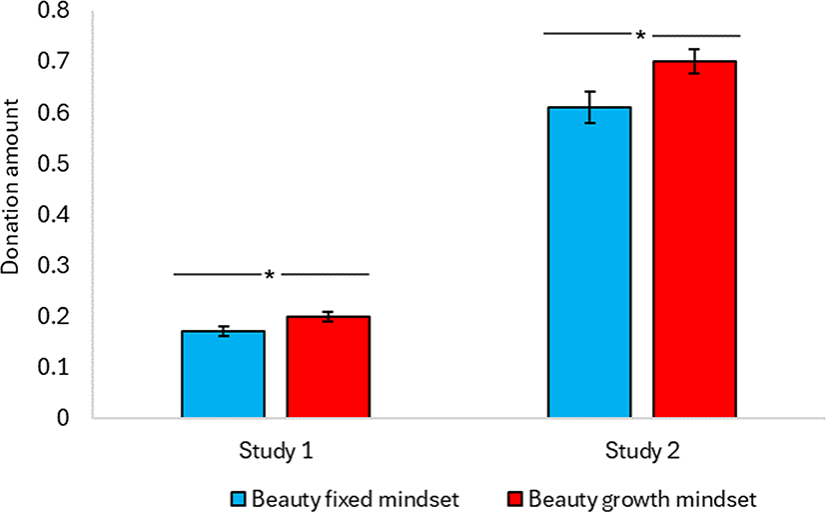
Donation amount. Donation amount as a function of beauty mindset in Studies 1 and 2. In Study 1, participants (*N* = 1,003) donated out of their €0.50 bonus, whereas in Study 2 (*N* = 214) the donation amount was the allocation out of €1.00 to a more other-empowering charity organization. *P < .05. Error bars denote standard errors.

**Fig. 2:**
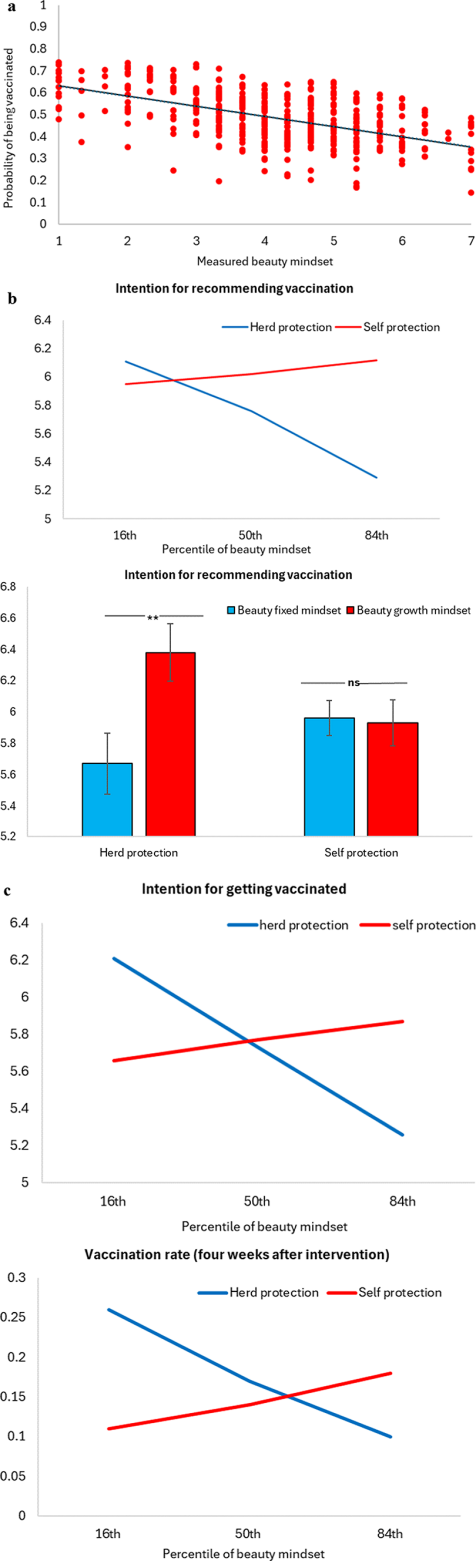
Vaccination rates and behavioral intentions in Studies 3 to 6 (total *N* = 1,693). (**a**) The relationship between measured beauty mindset and the likelihood of being vaccinated (Study 3, *N* = 429). (**b**) Intention for recommending vaccination to others. Beauty mindset was measured (Study 4, *N* = 410; top) or experimentally varied (Study 5, *N* = 424, error bars denote S.E.; bottom). (**c**) Vaccination rates and intention. Top, vaccination intention (Study 6, *N* = 430). Bottom, vaccination rates 4 weeks after exposure to intervention messages (Follow-up study of Study 6). For measured beauty mindset, higher (lower) number means higher tendency of holding a beauty fixed (growth) mindset (**a**,**b top**, and **c**). **P = .003; ns = non-significant.

Figures 1 and 2 present results on real donation behavior and responses to interventions for promoting COVID-19 vaccination obtained in Studies 1 through 6. Table 1 gives an overview and research questions addressed in each experiment. Manipulation checks, data exclusion information, and robustness checks of all studies are reported in Supplementary Information.

**Table 1:** Overview of design and questions addressed of all studies.

Study	Question of interest	Prosocial/altruistic behavior	Beauty/beauty mindset; and other treatment types
Study 1	Effect of beauty growth mindset on real donation	Monetary donation	(1) Beauty mindset experimentally varied
Study 2	Effect of beauty growth mindset on real donation	Monetary donation to an other-empowering charity organization	(1) Beauty mindset experimentally varied
Study 3	Correlational relationship between trait beauty mindset and current vaccination status	Current COVID-19 vaccination status	(1) Beauty mindset measured
Study 4	Effect of beauty growth mindset on responses to vaccination intervention messages	Intention for recommendation of vaccines to others	(1) Beauty mindset measured (2) Intervention messages (herd protection focus vs. self protection focus)
Study 5	Effect of beauty growth mindset on responses to vaccination intervention messages	Intention for recommendation of vaccines to others	(1) Beauty mindset experimentally varied (2) Intervention messages (herd protection focus vs. self protection focus)
Study 6	Effect of beauty growth mindset on responses to vaccination intervention messages	Vaccination intention and intention for recommendation of vaccines to others	(1) Beauty mindset measured (2) Intervention messages (herd protection focus vs. self protection focus)
Follow-up study to Study 6	Responses to vaccination intervention messages 4 weeks after intervention exposure	Vaccination status	(1) Beauty mindset measured (2) Intervention messages (herd protection focus vs. self protection focus)
Study 7	Effect of beauty growth mindset (vs. fixed mindset vs. no mindset) on voting behavior	Voting intention	(1) Beauty mindset experimentally varied
Study 8	Effects of growth mindset of different human traits (morality vs. beauty)	Intention for signing a petition fighting against domestic violence	(1) Beauty mindset experimentally varied (2) Morality mindset experimentally varied
Study 9	Effects of growth mindset of different human traits (intelligence vs. beauty)	Intention for signing a petition fighting against domestic violence	(1) Beauty mindset experimentally varied (2) Intelligence mindset experimentally varied
Study 10	More process evidence: self-perception of making impact on others (as a mediator)	Intention for supporting a stigmatized identity	(1) Beauty mindset experimentally varied

### Study 1

In this study (preregistration: https://aspredicted.org/RPZ_LHW, 10th April 2022), our key outcome variable is real monetary donation to a charitable project at GoFundMe, a crowdfunding site that allows people to raise money to support a cause. The charitable project was about raising money to support the education of orphans in Uganda. Participants (*N* = 1,003) were from five countries (Australia, France, Mexico, the UK, and the US). We conducted a one-way ANOVA with beauty mindsets (growth mindset vs. fixed mindset) as the independent variable, and donation amount as the dependent variable. Results revealed that holding a beauty growth mindset significantly increased donation amount (*M*_growth_ = 0.20, SD = 0.22 vs. *M*_fixed_ = 0.17, SD = 0.21; *F*(1, 1001) = 5.85, *P* = .016, *η*^2^ = 0.01). Including covariates (e.g., previous donation preferences) in the analyses did not meaningfully change the results in this and other studies (SI S6).

As an additional analysis to examine the effect of beauty mindsets, we conducted a linear regression with beauty mindset manipulation check as the independent variable and donation amount as the dependent variable. Results revealed that a beauty growth- (vs. fixed-) mindset led to higher donation amount (*B* = − 0.01, *t*(999) = -3.18, *p* = .002; the effect is negative as a growth mindset is denoted by lower fixed belief score; see manipulation check items in SI S4).

### Study 2

The purpose of Study 2 was to examine our prediction that a beauty growth (vs. fixed) mindset could dispose people to exert positive impact on others through engaging in prosocial behavior. To the extent that a charity option is empowering to beneficiaries, people who hold a beauty growth- (vs. fixed-) mindset would be likely to choose it. Thus, our key outcome variable is real monetary donation allocation to a more (vs. less) orphan-empowering charity organization. A pretest among a separate group of 200 participants verified our assumption that one charity is more orphan-empowering than the other (SI S7). Participants (*N* = 214) were undergraduate students from a European university. A 2 × 1 (Beauty mindset: growth vs. fixed) ANOVA on the amount of money allocated to the more empowering charity revealed a significant main effect of beauty mindsets, with growth-mindset (vs. fixed) participants allocating more money to the more orphan-empowering charity A (*M*_growth_ = 0.70, SD = 0.25 cents vs. *M*_fixed_ = 0.61, SD = 0.31, *F*(1, 209) = 5.21, *P* = .023, *η*^2^ = 0.024). Including covariates (e.g., self-rated beauty, perceived importance of beauty, trait empathy, and mood) in the analyses did not meaningfully change the results in this and other studies (SI S8).

Results of Study 2 shed some light on the psychological process underlying the effect of beauty-growth mindset on altruistic behavior; participants with a growth mindset of beauty were more likely to choose a more other-empowering charity organization, providing initial evidence for our hypothesis that a growth (vs. fixed) mindset of beauty is more likely to dispose people to exert a positive impact on others. Indeed, we conducted a post-test among 200 participants on Prolific (59.5% females; *M* = 42.29, SD = 13.32) in which participants read about one of the two charities as in the main study, and indicated the extent to which they perceive the charity is making a positive change for the beneficiaries (e.g. “This charity program makes a positive impact on the beneficiaries”). Results showed that the more-empowering charity (*M* = 5.40, SD = 1.32) was indeed perceived as making a higher level of positive impact on the beneficiaries than does the less-empowering charity (*M* = 5.02, SD = 1.13; *t*(198) = 2.22, *P* = .027).

### Study 3

This study was intended to examine if people’s trait beauty mindset is correlated with their COVID-19 vaccination behavior (i.e., had already been vaccinated: yes vs. no). Participants were from Mexico (*N* = 429). Our key outcome measure is the correlational relationship between vaccination rate and trait beauty mindset (measured).

We conducted a logistic regression with beauty mindsets and control variables (including, COVID-19 infection history, friends/family infection history, presence of incentive program for the COVID-19 vaccination in participants’ area/state, perceived vulnerability of getting infected, perceived vaccine efficacy, self-rated beauty, and perceived importance of beauty) as independent variables (see Methods and SI) and whether or not participants had been vaccinated as the dependent variable (Yes = 1, No = 0). Results revealed a significant correlational relationship between trait beauty mindsets and current vaccination status. Those who hold a growth mindset were more likely to indicate that they had been vaccinated (*Β* = − 0.16, SE = 0.08, Wald χ^2^ = 4.55, *P* = .033). One control variable, participants’ self-rated beauty had a significant positive relationship with current vaccination status (Β = 0.09, SE = 0.04, Wald χ^2^ = 7.55, *P* = .006). All other control variables had no significant effect (*P*s > 0.05) (Fig. 2a).

Results suggest that trait growth mindset is positively and significantly associated with vaccination status. However, it is unclear why this is the case and whether vaccination behavior is an act of prosocial behavior. In Studies 4 to 6, we investigated that. To do this, we designed and tested two intervention messages: one focusing on achieving herd protection against COVID-19 through vaccination, and one focusing on protecting the self through vaccination. To test the impact of beauty mindsets on prosocial behavior, we examined the role of beauty mindsets in the persuasive impact of these intervention messages (herd-protection focus vs. self-protection focus) on vaccination behavior.

### Study 4 and study 5

Getting vaccinated is an act of prosociality that can be beneficial to others (especially those who are not vaccinated) and the self. As noted earlier, we developed two intervention messages for motivating COVID-19 vaccination: protecting the herd vs. protecting the self (SI S10). If beauty growth mindset motivates prosocial behavior, participants in the growth (vs. fixed) mindset condition should be more persuaded by the herd-protection message.

Participants in Study 4 (preregistration: https://aspredicted.org/DNL_KVB, 8th March 2021) were from France (*N* = 410) and those in Study 5 were from the US (*N* = 424). We conducted Study 4 in August 2021 and Study 5 in June 2021. Although there was skepticism about the possibility to achieve herd immunity in the US and some other countries later on, by the time we conducted the studies, there were still discussions that herd immunity could be achieved by vaccination in the US^63^ and France^64^. Moreover, during these periods, the vaccination rates in these two countries were already high (64.5% and 89% respectively). Thus, we did not conduct a follow-up study on participants’ actual vaccination rate one month after their exposure to the intervention messages. However, we did such a follow-up for Study 6.

The experimental design of Studies 3 to 5 was almost identical except that chronic trait beauty mindsets were measured in Studies 3 and 4 whereas they were experimentally varied in Study 5. The key outcome variables in Studies 4 and 5 are behavioral intention to (1) get vaccinated (SIs S11 and S10), and (2) recommend the vaccine to others (Fig. 2).

Results showed a significant interactive effect of beauty mindsets and intervention messages in both Study 4 (*B* = − 0.42, *SE* = 0.14, *t* = -3.01, *P* = .003) and Study 5 (*F*(1, 419) = 5.07, *P* = .025, *η*^2^ = 0.01). Simple-effects tests showed that when the intervention message was focused on herd protection, growth-mindset theorists demonstrated higher intention to recommend the vaccine to others compared to fixed-mindset theorists (Study 4: *B* = − 0.35, *SE* = 0.10, *t* = -3.51, *P* = .0005; Study 5: *M*_growth/herd protection_ = 6.38, *SD* = 1.11 vs. *M*_fixed/herd protection_ = 5.67, *SD* = 2.03; *F*(1, 419) = 9.22, *P* = .003), whereas when the message was focused on self-protection, this effect disappeared (Study 4: *B* = 0.07, SE = 0.10, *t* = 0.73, *P* = .468; Study 5: *M*_growth/self protection_ = 5.93, *SD* = 1.56 vs. *M*_fixed/self protection_ = 5.96, *SD* = 1.87; *F*(1, 419) = 0.02, *P* = .891). Results of Studies 4 and 5 supported our predictions that participants with a beauty growth mindset (either as a trait or when it is experimentally manipulated) are more persuaded by a herd-protection intervention message than those with a beauty fixed mindset (Fig. 2b).

One may argue that the interactive effects observed in these studies were driven by a negative impact of a beauty fixed-mindset rather than a beauty growth-mindset, particularly if the self-protection message condition serves as some sort of a control. This is plausible. To directly examine whether the effect on prosocial behavior was driven by the effect of a growth- or a fixed-mindset, we conducted Study 7 in which a control condition was included.

Participants who answered No to our question of whether they had already been vaccinated were asked to indicate their intention to get vaccinated. As a majority of participants from France (85.9%; Study 4) and from the US (74.3%; Study 5) indicated that they had already got vaccinated, we did not conduct analyses on this dependent variable from these two samples. In Study 6, we examined that.

### Study 6

Participants were from Australia (*N* = 430). We conducted the study in July 2021, during which the dialogue on herd immunity was quite positive in Australia^65^. At that time, the country’s vaccination rate was 25%. This allowed us to conduct a follow-up study one month after they were exposed to our intervention (i.e., August 2021). Our key outcome measures are (1) participants’ intention for getting vaccinated, (2) intention for recommending COVID-19 vaccination to others (measured immediately after exposure to our intervention message), and (3) their vaccination status *one month* after exposure to our intervention message (measured in our follow-up study). The former two measures were administered immediately after the exposure to the intervention messages.

A linear regression treating the intention for getting vaccinated as the dependent variable, trait mindset (measured, mean-centered) and intervention messages (herd protection versus self protection) as independent variables was conducted. Results revealed a significant interaction of beauty mindsets and intervention message (*Β* = − 0.44, *SE* = 0.15, *t* = -2.97, *P* = .003). A moderation analysis showed that when the intervention message was focused on herd protection, growth (vs. fixed) mindset increased the intention of getting vaccinated (*Β* = − 0.36, *SE* = 0.11, *t* = -3.40, *p* < .001), whereas the effect was not significant when the message was about self protection (*Β* = 0.08, *SE* = 0.10, *t* = 0.77, *P* = .439) (Fig. 2c). Other comparisons were non-significant.

A similar analysis on participants’ intention for recommending COVID-19 vaccination to others was conducted. Importantly, results revealed a significant 2-way interaction (Fig. 2c) (*Β* = − 0.26, *SE* = 0.12, *t* = -2.20, *P* = .028). A moderation analysis (Hayes’ Process model 1) showed that when the intervention message was focused on herd protection, participants in the growth mindset (vs. fixed mindset) condition were more likely to recommend the vaccines to others (*Β* = − 0.24, *SE* = 0.08, *t* = -2.92, *P* = .004), whereas the difference was not significant when the intervention message was focused on self protection (*Β* = 0.02, *SE* = 0.08, *t* = 0.21, *P* = .836).

### Follow-up to study 6

One month after exposure to the intervention message, we conducted a follow-up study by asking participants to report their new vaccination status (“Have you been vaccinated for COVID-19?”, yes/no). 73.7% of the sample (*N* = 317) who indicated that they had not been vaccinated were followed up on their subsequent, actual vaccination behavior one month after this survey was conducted, out of which 291 participants responded. A logistic regression showed a significant interaction of beauty mindsets and intervention message on subsequent, actual vaccination behavior (*B* = 0.65, *SE* = 0.26, Wald χ^2^ = 6.45, *P* = .014). Further moderation analysis showed that in the herd-protection message conditions, growth (vs. fixed) mindset led to higher vaccination rate (*B* = − 0.43, *SE* = 0.18, *P* = .010). In the self-protection message conditions, the effect of beauty mindsets on vaccination rate was not significant (*P* = .237) (Fig. 2b,c).

### Study 7

Studies 7 to 10 were intended to examine the cognitive underpinnings of the effects we observed. In particular, we examined several alternative accounts and the psychological mechanism underlying the effect of beauty growth mindset on altruistic behavior.

In Study 7, we aimed to (1) investigate whether a growth mindset of beauty increased prosocial behavior or a fixed mindset of beauty reduced it by including a control condition and (2) examine a different prosocial behavior, namely voting^66^.

Participants were recruited from the US (*N* = 374; 48.7% females, *M*_age_ = 36.84, SD = 13.04) at Cloud Research. Our key outcome variable is participants’ intention for voting in the upcoming presidential election in 2020. Voting intentions were measured with two items: “How likely that you will register to vote?” and “How likely that you will vote?” (1 = not likely at all, 7 = very likely; α = 0.85). A 3 × 1 ANOVA on voting intentions revealed a significant effect of beauty mindsets (*F*(2, 371) = 4.82, *P* = .009, *η*^2^ = 0.025). Further, a post-hoc Tukey’s HSD test showed that participants in the beauty growth-mindset condition exhibited higher voting intentions compared to participants in the beauty fixed-mindset condition (*M*_growth_ = 6.42, SD = 0.95 vs. *M*_fixed_ = 5.86, SD = 1.84; *P* = .010) and the control condition (*M*_growth_ = 6.42, SD = 0.95 vs. *M*_control_ = 5.97, SD = 1.60; *P* = .050). The difference between the beauty fixed mindset and control conditions was not significant (*P* = .837).

### Studies 8 and 9

One may argue that it is the malleability nature of growth mindset in general rather than the mindset of beauty driving the results we observed thus far. To examine the validity of this alternative account, in Studies 8 and 9, we tested whether the effect of mindsets on prosocial behavior was specific to beauty domain or not.

We examined the proposed effects in beauty and morality domain respectively in Study 8. Moreover, we examined this alternative account in beauty and intelligence domain respectively in Study 9 (preregistration: https://aspredicted.org/ZHR_RSG, 11th May 2023). We did not have any a priori prediction about the effects of morality or intelligence mindsets on engagement in prosocial behavior. Past research has shown that moral identity and setting mastery goals may promote prosocial behavior^67,68^. However, there is also evidence that moral identity reduced charity giving^69^, and that morality is related to moral condemnation or moralistic punishment of others^70^ presumably because there are qualifications (e.g., judgment of justice) which govern when morality leads to other-benefiting behavior. Thus, being moral does not directly lead to exerting a positive impact on others. Moreover, the effects of morality and intelligence growth-mindset on engagement in prosocial behavior have been mixed^71–75^. For example, there has been evidence showing that morality growth-mindset holders are more tolerant towards one’s immoral actions^76^.

Our key outcome measure in Studies 8 and 9 is participant’s willingness to sign a petition for an important cause, fighting against domestic violence.

*Study 8 results*: A 2 (Mindset: growth vs. fixed) × 2 (Domain: beauty vs. morality) ANOVA on willingness to sign the petition showed that the main effects of mindsets and domain were not significant (*P*s > 0.2). However, there was a significant interaction of mindsets and domain (*F*(1, 378) = 5.07, *P* = .025, *η*^2^ = 0.013). Follow-up simple-effects tests revealed that in the beauty domain, participants in the growth (vs. fixed) mindset condition were more willing to sign the petition (*M*_growth/beauty_ = 5.98, SD = 1.26 vs. *M*_fixed/beauty_ = 5.45, SD = 1.67; *P* = .013). In contrast, this difference was not significant in morality (*M*_growth/morality_ = 5.65, SD = 1.79 vs. *M*_fixed/morality_ = 5.82, SD = 1.35; *P* = .459).

*Study 9 results*: A 2 (Mindset: growth vs. fixed) × 2 (Domain: beauty vs. intelligence) ANOVA on willingness to sign the petition revealed a significant main effect of mindsets, with participants in the growth (vs. fixed) mindset condition indicating greater willingness to sign the petition (*M*_growth_ = 5.40, SD = 1.60 vs. *M*_fixed_ = 4.89, SD = 1.84, *F*(1, 389) = 8.59, *P* = .004, *η*^2^ = 0.02), a non-significant main effect of domain (*M*_beauty_ = 5.22, SD = 1.62 vs. *M*_intelligence_ = 5.07, SD = 1.86, *F*(1, 389) = 0.79, *P* = .374, *η*^2^ = 0.002), and importantly, a significant interaction effect of mindsets and domain (*F*(1, 389) = 4.16, *P* = .042, *η*^2^ = 0.01). Follow-up simple-effects tests further showed that in the beauty domain, growth (vs. fixed) mindset theorists indicated greater willingness to sign the petition (*M*_growth/beauty_ = 5.65, SD = 1.26 vs. *M*_fixed/beauty_ = 4.79, SD = 1.82, *F*(1, 389) = 12.45, *P* < .001, *η*^2^ = 0.03). In contrast, in the intelligence domain, there was no difference in willingness to sign the petition (*M*_growth/intelligence_ = 5.14, SD = 1.86 vs. *M*_fixed/intelligence_ = 4.99, SD = 1.87, *F*(1, 389) = 0.39, *P* = .531, *η*^2^ = 0.001).

In both Studies 8 and 9, we found significant effects of beauty mindset on participants’ intention for exerting positive social impact (a form of prosocial behavior). We do not observe any significant effects of intelligence or morality mindsets on the dependent variable. As noted earlier, past research on these latter effects have been mixed^71–75^. We speculate that effects of mindsets are likely to depend on the domains in which they are studied^72,77^.

### Study 10

In this study, we aimed to demonstrate that perceived ability to make a positive social impact mediates the effect of beauty growth mindset on altruistic behavior. We investigated a different prosocial behavior—supporting a stigmatized identity. The key outcome measure is participants’ behavioral intention for supporting a LGBTQ+ owned business through purchasing products from it, telling others about this store, and donating to the store to show support (1 = not likely at all, 7 = very likely; α = 0.91).

An ANOVA revealed a significant effect of beauty mindsets, with beauty growth (vs. fixed) mindset participants showing greater support for the business (*M*_growth_ = 3.56, SD = 1.73 vs. *M*_fixed_ = 2.99, SD = 1.60; *F*(1, 322) = 9.32, *P* = .002, *η*^2^ = 0.03). More importantly, the perception of the ability to make a social impact significantly mediated the effect of beauty growth mindset on supporting the stigmatized identity (*β* = 0.19, SE = 0.07, 95% CI = [0.07, 0.34]).

## Discussion

The present research demonstrates that beauty growth-mindset could bring profound societal effects. Previous research has largely focused on the social benefits beauty brings. Using 10 experiments involving 4,449 participants from five countries (including the US, the UK, France, Mexico, and Australia), we showed that beauty growth-mindset could motivate prosocial behavior. Our results consistently showed that a growth-mindset of beauty (vs. fixed-mindset vs. control condition) disposes people to behave altruistically—making significantly more real monetary donation to charity, allocating significantly more real monetary donation to an other-empowering charity, being more likely to get a COVID-19 vaccination when exposed to an intervention message focusing on achieving herd protection (vs. self-protection), and supporting various important causes. Moreover, we examined the psychological mechanism underlying the effects of beauty mindsets on prosocial behavior. Study 2 revealed that participants in the growth- (vs. fixed-) mindset condition were more likely to donate more money to a more (vs. less) empowering charity project. Study 8 and 9 ruled out two key alternative accounts that (1) growth- (vs. fixed) mindsets per se, and (2) mindsets of other aspects of human attributes such as morality and intelligence could produce the effects on prosocial behavior observed across our studies. Study 10 showed that the effect of beauty mindsets on prosocial behavior was indeed mediated by a heightened perception of the ability to exert positive social impact among participants with a beauty growth- (vs. fixed-) mindset. Our findings offer key contributions to beauty, altruism, and implicit theories literature. We provide evidence that beauty mindsets could translate into altruistic behavior. Extensive empirical work in recent decades provided evidence for the presence of beauty-is-good stereotype in social judgment and behavior. Most of these studies have focused on how attractive people are evaluated and the benefits they enjoy. However, research on the effects of beauty on perceptions of kindness and altruistic behavior (i.e., beautiful people are altruistic) are sparse, limited, and inconsistent. As noted earlier, most studies on the beauty-is-good stereotype were focused on good aspects such as perceptions of intelligence, social competence, reproductivity, and the social benefits they obtain. Only a handful of studies examined the relationship between beauty and kindness. However, meta-analyses have shown that beauty does not lead to perception of kindness and concern for others^27,30,31^. Departing from this line of research, the current research focuses on how people understand beauty (i.e., beauty mindsets) and how beauty mindsets shape prosocial and altruistic behavior. The present research confirmed that beauty mindsets that describe *how humans personally relate to beauty* matter. Extensive research has shown that implicit theories have robust impact on how people make sense of the world and assess the self and others. We investigate whether such an intimate link exists in the context of beauty.

How do we promote prosocial behavior using beauty? Our studies showed that people who (either chronically or incidentally) hold a growth-mindset of beauty will respond positively to interventions focusing on radiating a positive impact on others. Given past research findings, beauty does not seem to directly translate into altruistic behavior. Our research suggests that an effective way for eliciting this effect would be to design an intervention for activating the beauty growth-mindset. Participants in the beauty growth mindset conditions were more persuaded by an intervention focusing on achieving herd protection than by one focusing on achieving self-protection, as evidenced by an increase in their intention to get vaccinated immediately after exposure to the intervention message, and their vaccination status one month after the experiment. As a starting point, the present work is the first to investigate the impact of beauty growth-mindset on prosocial behavior. We provided strong evidence for the effect of beauty growth-mindset on various types of altruistic behavior across various settings and across several countries. Study 1 examined real altruistic behavior on an online charity project. Real donations were involved in Studies 1 and 2. We considered real-life settings—Studies 3 to 6 tested the persuasive impact of two intervention messages—one focusing on getting vaccinated to protect the herd and the other focusing on getting vaccinated to protect the self. Results showed that beauty growth mindset plays a role in the persuasive impact of these interventions on self-reported vaccination behavior. Studies 7 to 10 concerned the intention for supporting important causes through voting, signing a petition to fight against domestic violence, and supporting a stigmatized identity. Future research should examine that in the field.

In summary, the present research tested the effect of beauty on altruistic behavior via beauty mindsets. The impact of beauty on altruism has been argued by philosophers, scientists, and laypeople for centuries. Our findings advance theoretical understanding of the causal link from beauty growth mindset to prosocial behavior. Given the well-established robustness of the deep-seated effects of mindsets on behavior in both lab and field, extending the present study to the field for promoting altruistic behavior is a promising and important future work direction.

## Methods

The research was approved by the Institutional Review Board of the Chinese University of Hong Kong, Shenzhen, and the Nova School of Business and Economics. All methods were performed in accordance with the relevant guidelines and regulations. Informed consent was obtained from participants prior to participating in the experiments. As we did not know the effect sizes before conducting the studies, we have at least 100 participants in each condition based on the recommendation on how to select appropriate sample sizes in the literature^78^.

### Study 1

A total of 1,003 participants from five countries, including Australia, France, Mexico, the UK, and the US, were recruited from Prolific. This study is preregistered. Participants were randomly assigned to one of the two conditions of a one-factor (beauty mindset: growth mindset vs. fixed mindset) between-subjects design. To manipulate beauty mindsets, we adapted a well-established manipulation in the literature of mindsets in the intelligence domain^52^ by having participants read an article which describes scientific findings that beauty is malleable or fixed (see stimuli and manipulation checks in SI S2 and SI S4).

Our preregistered outcome variable is monetary donation amount. We told participants that we were soliciting donation for a GoFundMe project which supports the education for orphans in Uganda. Participants were told that: “As researchers, we try to help people who are in need. This month, we are soliciting donation to send to a GoFundMe project which supports the education for orphans in Uganda. The details of this GoFundMe project can be found in the link below; please take a look (participants were shown the link to click on). Today, we would like to ask you if you want to donate to this charity. Any amount will help. We will make the total donation to the project on your behalf. At the end of this study, we will give you a bonus of £0.50. If you agree to donate, we will take the amount you want to donate from this bonus; you will then receive the rest”. Participants then indicated whether they would like to donate to the GoFundMe project (yes/no; results in SI S5) and the amount they would like to donate from their bonus (out of £0.50).

In Studies 1 and 2, several control variables, including participants’ frequency of donating to charities, amount of donation made in the past, and attitude towards related charities were considered (SIs S6 and S8).

### Study 2

To test whether a growth mindset of beauty promotes the choice of making a positive impact on others, we examine participants’ behavior of choosing an other-empowering charity program. We considered real donation allocation behavior as the dependent variable.

We recruited 214 undergraduate students (55.1% females, *M*_age_ = 22.94, SD = 1.35) from a large European school who participated in the study for course credit. Participants were randomly assigned to one of the two conditions in a one-factor (Beauty mindset: growth mindset vs. fixed mindset) between-subjects design.

After the manipulation of beauty mindsets, as a “separate” study, participants were shown information of two donation options: “As researchers, we work with organizations to help people who are in need. This year, we are in a partnership with two organizations to help unfortunate orphans who are living in extreme poverty in Africa. For confidentiality purposes, we refer to the two organizations as Charity A and Charity B”. Among the two options, charity A was designed to be more empowering: “Charity A: who use donations to provide programs in Kenya and Uganda to equip orphans with tools and training to overcome life-threatening poverty”, compared to charity B: “who use donations to buy food and necessities for poor orphans in Kenya and Uganda to overcome life-threatening poverty”. A pretest among a separate group of 200 participants verified our assumption that charity A is more empowering than charity B (SI S7).

We captured participants’ choice for the empowering charity with a real behavior measure. We informed participants that “to thank you for your participation in this research, our research team will donate 1 euro to charities on your behalf”. On the desk for each participant, we provided 5 coins of 20 cents, totaling 1 euro, and two envelopes. We asked participants to allocate 1 euro to each charity (A and B) in each envelope.

After that, several control variables, including self-rated beauty, importance of beauty, feelings of empathy and mood were measured (see detailed rationale for including these control variables in SI S8). Lastly, manipulation checks were administered (SI S4).

### Study 3

A total of 429 participants from Mexico (46.2% females, *M*_age_ = 26.57, SD = 7.69) were recruited from Prolific. Participants were told that they would be asked some information about COVID-19 vaccination. Participants indicated whether they have been vaccinated for COVID-19 (yes/no). Chronic disposition of beauty mindsets was measured. Specifically, we adopted Faust et al.’s^21^ measure of beauty mindsets with three items: “You have a certain amount of beauty and you can’t do much to change it”, “Your beauty is something about you that you can’t change very much”, and “You can enhance your appearance, but you can’t really change your basic beauty” (1 = strongly disagree, 7 = strongly agree; α = 0.83). An average of the three items serves as implicit theories index, with the lower score indicating greater growth mindset^43^. These items are the same as the manipulation check items that we used in previous studies where we manipulated beauty mindsets.

In addition to measuring participants’ chronic beauty mindset and their current vaccination status, we included several control variables that may influence vaccination behavior, including: “Have you ever got tested positive for COVID-19?”; “Has anyone you know got COVID-19?”, and “Is there any incentive program for the COVID-19 vaccination in your area/state?” (1 = yes, 0 = no). Further, we measured perceived vulnerability^79^ with three items: “How likely do you think you are to contract COVID-19 over the next month?” (1 = very unlikely; 7 = very likely); “In the last week, how often have you worried about catching COVID-19?” (1 = never, 7 = all the time)”; “What do you think your chances of getting COVID-19 over the next month are compared with others outside your family?” (1 = no chance, 7 = certain); and response efficacy^79^ with two items: “Vaccination is a very effective way to protect people against COVID-19”, “Vaccination greatly reduces the risk of catching COVID-19” (1 = strongly disagree, 7 = strongly agree). Finally, we included measures for perception of one’s own beauty and perceived importance of beauty as in Study 2 (SI S8).

### Study 4

A total of 410 (55.4% females, *M*_age_ = 26.65, SD = 8.55) were recruited from France on Prolific. They were randomly assigned to a one-factor, two-level, 2 (Intervention message focus: herd protection vs. self-protection) × 1 between-subjects design. This study is preregistered.

Participants were told that they would be shown some information about COVID-19 vaccine and asked to answer a few questions about it. In the herd-protection message condition, for example, participants read: “what we know about coronavirus so far suggests that we would need at least 70% of the population to be immune to keep the rate of infection down without restrictions on activities. The fastest way to achieve herd immunity is for each of us to do our part to reduce the spread of the virus; that is, to get vaccinated as soon as a vaccine becomes available to us”. Participants in the self-protection message condition read that “COVID-19 vaccines are effective at protecting you from getting sick, especially severe illness and death. Based on what we know about COVID-19 vaccines, people who have been fully vaccinated can start to resume activities that they did prior to the pandemic…Thus, get vaccinated as soon as a vaccine becomes available to protect yourself.” We conducted studies (i.e., Studies 3 to 6, and Follow-up of Study 6) using the COVID-19 vaccination context in several countries. To enhance realism, the messages were adapted in each country to be aligned with the actual statistics and COVID-19 regulations in each country (SI S10).

We measured participants’ current vaccination status (“Have you been vaccinated for COVID-19?”, yes/no) and their decision of recommending the vaccine to others (“How likely are you to recommend others to get the vaccine?”, 1 = not likely at all, 7 = very likely). Chronic disposition of beauty mindsets was measured with the same items as in Study 3. The same control variables were measured as in Study 3.

### Study 5

A total of 424 participants from the US (52.4% females, *M*_age_ = 34.24, SD = 12.30) were recruited from Prolific. They were randomly assigned to one of the four conditions in a 2 (Beauty mindset: growth mindset vs. fixed mindset) × 2 (Intervention message: herd protection vs. self-protection) between-subjects design.

Beauty mindsets were manipulated as in Studies 1 and 2. After the manipulation, as a “separate” study, participants were told that they would be shown some information about COVID-19 vaccine and asked to answer a few questions about it as in Study 4 (SI S10). That is, they were shown the intervention messages (herd-protection vs. self-protection).

Vaccination status, the intention of recommending the vaccine to others, and the same control variables were measured as in Studies 3 and 4.

### Study 6

A total of 430 participants from Australia (51.2% females, *M*_age_ = 33.73, SD = 11.84) were recruited from Prolific and were randomly assigned to a one-factor, two-level, 2 (Intervention message: herd protection vs. self-protection) × 1 between-subjects design. Participants were told to view some information about COVID-19 and asked to answer a few questions about it as in Studies 4 and 5 (SI S10). That is, they were shown the intervention messages (herd-protection vs. self-protection). Participants’ chronic disposition of beauty mindsets was also measured.

Immediately after exposure to the intervention, they indicated their vaccination status and the intention of recommending the vaccine to others. The same control variables were measured as in Studies 3 to 5.

### Follow-up to study 6

At the time we conducted these studies in Australia (Study 6; conducted in July 2021 and followed up in August 2021), France (Study 4; conducted in August 2021) and the US (Study 5; conducted in June 2021), we were aware that the proportions of individuals getting vaccinated were increasing, especially in France and the US. The overall country’s vaccination rate was 25%, 64.5%, and 89% in Australia, France, and the US respectively. Thus, we followed up on participants’ subsequent vaccination behavior one month after they were exposed to our intervention only in the Australian sample (i.e., Study 6). Specifically, one month *after* we conducted the initial study, we conducted this follow-up study by asking participants to report their new vaccination status (“Have you been vaccinated for COVID-19?”, yes/no).

### Study 7

We recruited 374 US participants (48.7% females, *M*_age_ = 36.84, SD = 13.04) from Cloud Research. Participants were randomly assigned to one of the three conditions in a one-factor (Beauty mindsets: growth mindset vs. fixed mindset vs. control) between-subjects design.

We employed the same manipulation for beauty mindsets as in previous studies, with the addition of a control condition in which participants read an article about the properties of water^48^. Then, in an ostensibly unrelated task, we asked participants to think about the upcoming presidential election in 2020. Voting intentions were measured with two items: “How likely that you will register to vote?” and “How likely that you will vote?” (1 = not likely at all, 7 = very likely) (α = 0.85).

The same control variables administered in Study 2 and participants’ self-reported actual voter registration intention were considered. Further, in this study, we also controlled for long-term orientation, as it has been shown in past research to influence prosocial behaviors^80^.

### Study 8

One may argue that it is the malleability nature of growth mindset in general, rather than the mindset of a specific domain, driving the results observed thus far. To examine the validity of this alternative account, in Study 8, we tested whether the effect of mindsets on prosocial behavior was specific to beauty domain or not. We recruited 402 participants from Cloud Research (59.5% females, *M*_age_ = 30.32, SD = 9.45). Participants were randomly assigned to one of the four conditions in a 2 (Beauty mindset: growth mindset vs. fixed mindset) × 2 (Domain: beauty vs. morality) between-subjects design. We employed the same manipulation of mindsets as in previous studies and adapted it according to the morality domain (SI S18).

The main dependent variable was participants’ willingness to sign a petition which is a proxy for prosocial behavior. Specifically, we told participants that: “As researchers, we work with organizations who are trying to address different social challenges. This year, we are working with the International Domestic Violence Action Center, whose mission is to end domestic violence and other forms of abuse. At the moment, the Center is having an online petition to raise awareness about domestic violence and to gather support to shelters that provide housing for victims of domestic violence”. Participants then responded to the question: “How willing are you to sign the petition?” (1 = not at all, 7 = very much). The same control measures were administered as in Study 2 (SI S21).

### Study 9

In this preregistered study, we pitted mindsets of beauty against another domain, intelligence. In existing literature, there is no evidence of the link between intelligence mindsets and prosociality. We predict that an intelligence growth (vs. fixed) mindset would not influence prosocial behavior.

We recruited 400 participants from Prolific (47.8% females, *M*_age_ = 40.57, SD = 15.15). Participants were randomly assigned to one of the four conditions in a 2 (Beauty mindset: growth mindset vs. fixed mindset) × 2 (Domain: beauty vs. intelligence) between-subjects design.

We employed the same manipulation of mindsets as in previous studies. The article of intelligence mindsets was almost identical to the beauty articles, except that the domain was different (SI S19). Following the mindsets manipulation, we measured prosocial behavior in the same way as in Study 8; i.e. we measured participants’ willingness to sign a petition for domestic violence cause.

### Study 10

A total of 363 participants from Cloud Research (61.2% females, *M*_age_ = 36.65, SD = 8.74) participated in the study. They were randomly assigned to one of the two conditions in a one-factor (Beauty mindset: growth mindset vs. fixed mindset) between-subjects design.

We used the same manipulation of beauty mindsets as in previous studies. Then, in an ostensibly unrelated task, we presented participants information about a real LGBTQ+-owned business, as follows: “Muxe is one of the emerging LGBTQ+-owned businesses based in New York. Muxe offers t-shirts with various styles and designs. The brand’s inspiration is highlighted as follows: “In Zapotec cultures of Oaxaca, a Muxe is an assigned male at birth individual who dresses and behaves in ways otherwise associated with the female gender; they may be seen as a third gender”. The brand also contributes a portion of their funds to LGBTQ+ charities”. We showed participants a sample t-shirt from the brand, followed by a measure of support with three items: “How likely are you going to purchase from this brand?”, “How likely are you going to tell others about this brand?”, and “How likely are you going to donate to this store to support them?” (1 = not likely at all, 7 = very likely) (α = 0.91).

*Mediator* The measure of perception of ability to make a positive impact on others was adopted from the literature^81^, with: “I have the ability to shape outcomes in the world”, “I can determine what happens to people”, “I can have an effect on society”, and “I have influence in the world” (1 = strongly disagree, 7 = strongly agree) (α = 0.87).

Lastly, manipulation checks and the same control variables as in previous studies including perception of one’s own beauty, perceived importance of beauty, and mood were administered (SI S22).

## Electronic supplementary material

Below is the link to the electronic supplementary material.

Supplementary Material 1

## Data Availability

The research was approved by the Institutional Review Board of the Chinese University of Hong Kong, Shenzhen, and the Nova School of Business and Economics. Informed consent was obtained before collection of data in the experiments. The data analyzed and codes of this research can be accessed via this link (https://osf.io/n6xst/?view_only=4ad7a838fbd04acfb0921f04aced8213) and will be publicly available after the manuscript is accepted for publication.
