# Supplementary material for: Beauty growth-mindset promotes prosocial and altruistic behavior
Source: Sci Rep. 2024 Dec 4;14:30244. doi: 10.1038/s41598-024-82134-y (PMC11618811; doi:10.1038/s41598-024-82134-y)
Supplement: Supplementary file 1 — Supplementary Material 1 [file 41598_2024_82134_MOESM1_ESM.docx]

**Supplemental Information**

for

**Beauty growth-mindset promotes prosocial and altruistic behavior**

**S1. Deviations from preregistrations**

In Study 1, we preregistered that we would also explore whether or not the participant clicks on the link of the GoFundMe project. Due to technical issues, this data was not captured. In addition, although donation likelihood is *not* our preregistered outcome variable, it would be worth exploring the effect of beauty mindset on it, thus we conducted an analysis on this dependent variable (SI S5).

In Study 4, we preregistered the hypothesis that beauty growth- (vs. fixed) mindset theorists will be more likely to get vaccinated for covid-19 and recommend the vaccine to others when getting vaccinated is to achieve herd immunity (vs. to protect oneself). Only 56 participants had not been vaccinated; this sample was too small for us to analyze the results for likelihood to get vaccinated. Thus, in this study we only analyzed the data for the likelihood to recommend the vaccine to others.

In Study 9, there was no deviation from the preregistration.

**S2. Beauty mindset stimuli**


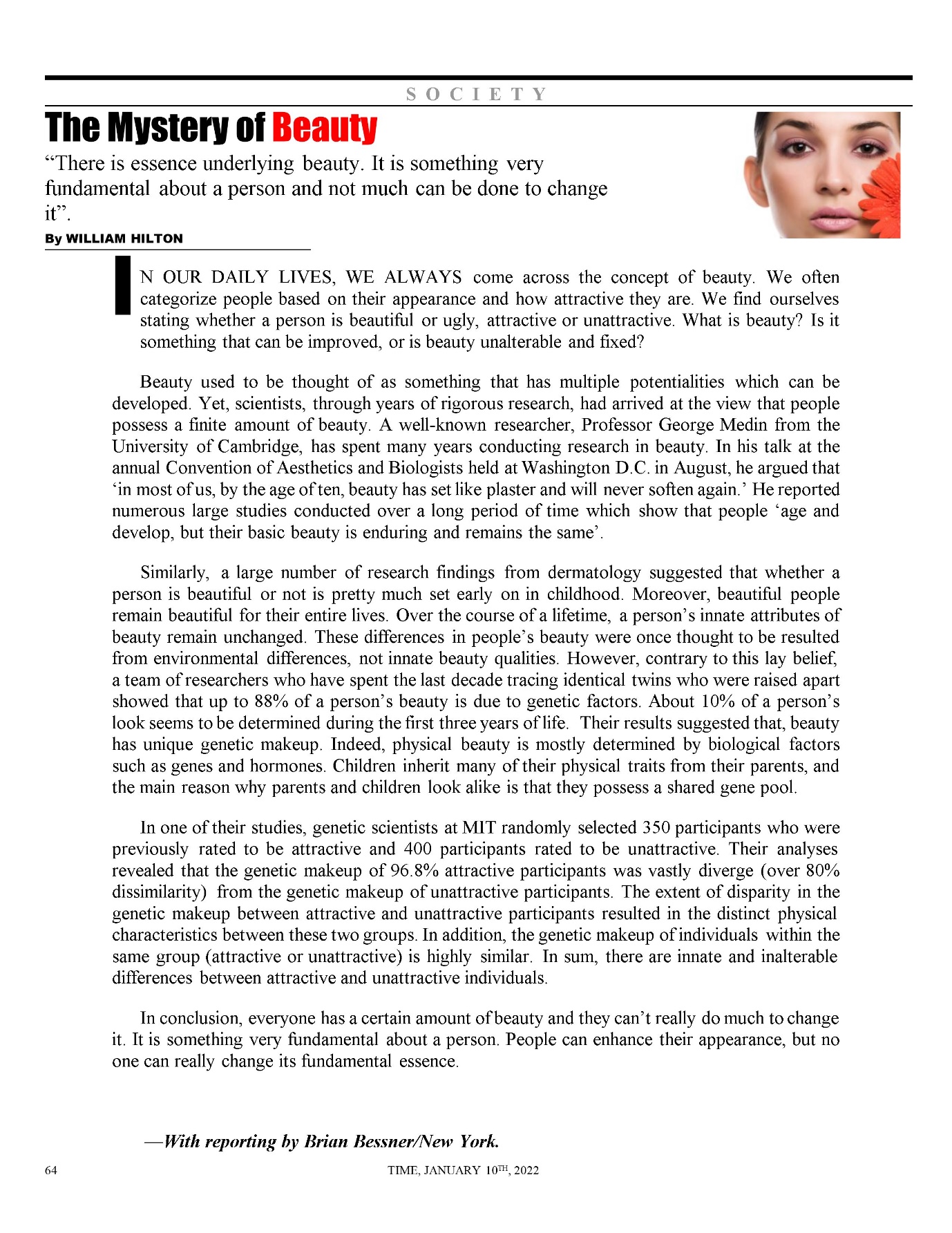


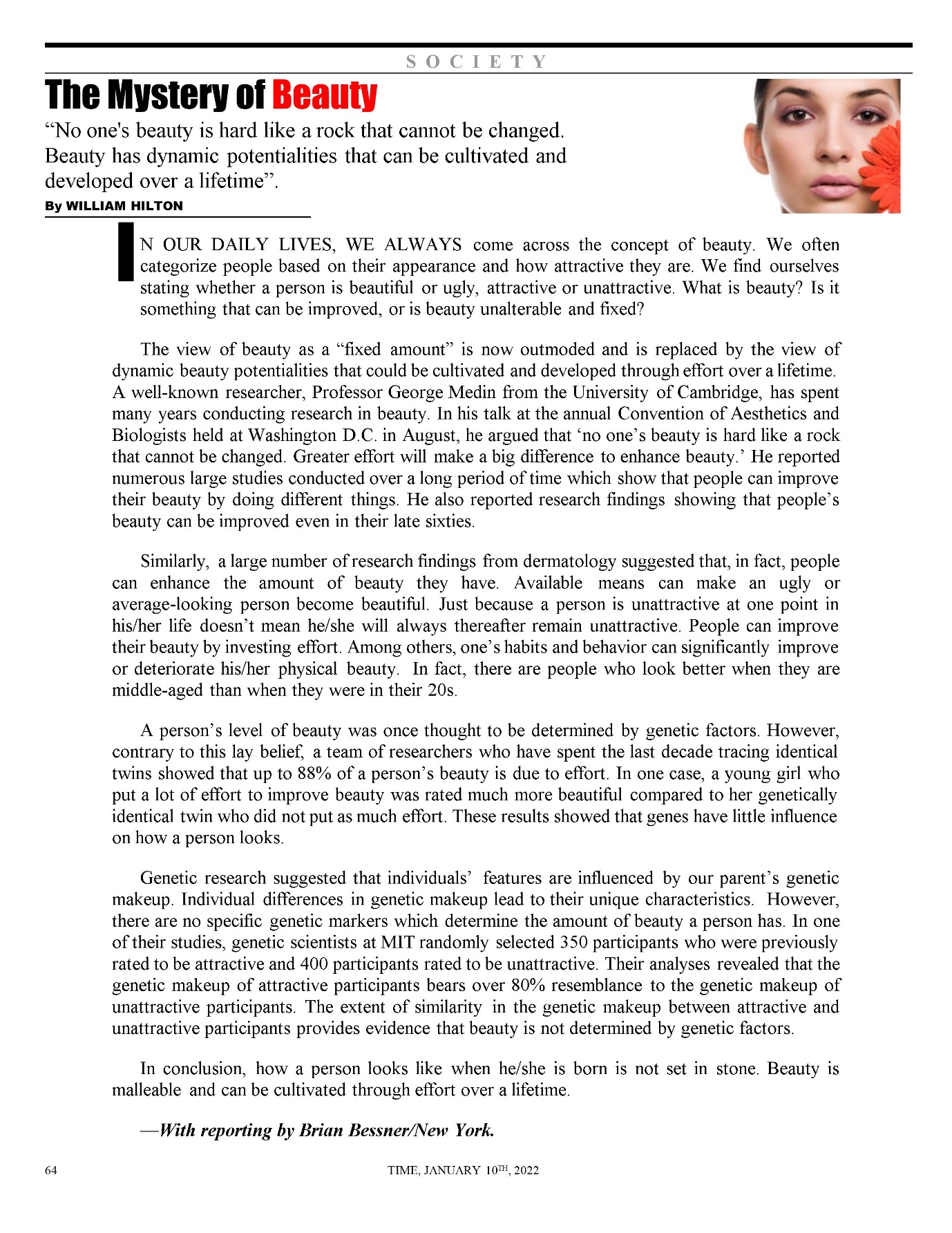


To reinforce the manipulation, participants were asked to write a summary of the main theme of the article and provide examples from their own experiences to support the theme of the article. This written response also served as an attention check (preregistered in Studies 1 and 9). We used this attention check throughout all studies in which we manipulated beauty mindsets.

**S3. Exclusions across studies**

**Study 1.** All participants provided a meaningful summary of the article (preregistered), thus no participant was excluded in this study.

**Study 2.** All participants provided a meaningful summary of the article, thus no participant was excluded in this study.

**Study 3.** No participant was excluded in this study.

**Study 4.** Five participants failed an attention check (“If you have read this question carefully, please do not respond to the scale below”, pre-registered) and thus were removed from the analysis.

**Study 5.** One participant failed to provide a meaningful summary of the article, and thus was removed. The final sample included 423 participants (52.2% females, *M*_age_ = 34.20, SD = 12.30).

**Study 6.** Two participants’ responses were incomplete, thus the final sample consisted of 428 participants.

**Study 7.** All participants provided a meaningful summary of the article, thus no participant was excluded in this study.

**Study 8.** Eighteen respondents did not provide a meaningful summary of the article, thus were removed. Further, two participants did not complete the study. Our final sample included 382 participants (59.6% females, *M*_age_ = 30.27, SD = 9.32).

**Study 9.** Seven participants failed to provide a meaningful summary of the article (preregistered), and thus were excluded. The final sample included 393 participants (48.3% females, *M*_age_ = 40.73, SD = 15.18).

**Study 10.** Thirty-nine participants failed to provide a meaningful summary of the article, and thus were removed. The final sample included 324 participants (63% females, *M*_age_ = 36.94, SD = 8.85).

**S4. Manipulation check of beauty mindset across studies**

In all studies (i.e., Studies 1, 2, 5, 7-10) where we manipulated beauty mindset, we included a manipulation check for our beauty mindset manipulation with three items, adopted from Faust et al.’s (2004) measures: (1) You have a certain amount of beauty and you can’t do much to change it; (2) Your beauty is something about you that you can’t change very much; (3) You can enhance your appearance, but you can’t really change your basic beauty (1 = strongly disagree, 7 = strongly agree).

**Study 1**. An independent t-test revealed that participants in the fixed (vs. growth) mindset condition indicated greater fixedness of beauty (*M*_fixed_ = 4.10, SD = 1.66 vs. *M*_growth_ = 3.78, SD = 1.50, *t*(999) = -3.17, *P* = .002).

**Study 2.** An independent t-test showed that participants in the fixed (vs. growth) condition indicated greater fixedness of beauty (*M*_fixed_ = 4.21, SD = 1.37 vs. *M*_growth_ = 3.39, SD = 1.44, *t*(212) = 4.26, *P* < .001). Thus, our beauty mindset manipulation was successful.

**Study 5**. A 2 (Beauty mindset: fixed vs. growth) × 2 (Intervention message: herd protection vs. self protection) ANOVA on the perceived fixedness of beauty revealed only a significant main effect of beauty mindset; participants in the fixed (vs. growth) mindset indicated greater fixedness of beauty (*M*_fixed_ = 4.16, SD = 1.66 vs. *M*_growth_ = 3.31, SD = 1.41; *F*(1, 416) = 32.88, *P* < .001). The main effect of intervention message (herd vs. self protection) was not significant (*P* > .4), and the interaction effect of beauty mindset and intervention message was non-significant (*P* > .1). Thus, our beauty mindset manipulation was successful.

**Study 7.** A 3 (Beauty mindset: growth vs. fixed vs. control) ANOVA on the fixedness of beauty revealed a significant effect of beauty mindset (*F*(2, 370) = 9.88, *P* < .001). A post-hoc Tukey's HSD test showed that participants in the growth mindset condition indicated lower fixedness of beauty compared to the fixed mindset condition (*M*_growth_ = 3.40, SD = 1.55 vs. *M*_fixed_ = 4.24, SD = 1.66; *P* < .001) and the control condition (*M*_growth_ = 3.40, SD = 1.55 vs. *M*_control_ = 4.06, SD = 1.52; *P* = .003). Thus, our manipulation of beauty mindset was successful.

**Study 8.** A 2 (Beauty mindset: growth vs. fixed) × 2 (Domain: beauty vs. morality) ANOVA on the fixedness of beauty (vs. morality) revealed a significant main effect of beauty mindset (*F*(1, 371) = 35.75, *P* < .001) with fixed (vs. growth) mindset showing higher fixedness (*M*_fixed_ = 4.51, SD = 1.31 vs. *M*_growth_ =3.70, SD = 1.47), a significant main effect of domain (*F*(1, 371) = 6.64, *P* = .01) with higher fixedness in beauty (vs. morality) (*M*_beauty_ = 4.26, SD = 1.30 vs. *M*_morality_ = 3.93, SD = 1.59), and a significant interaction of beauty mindset and domain (*F*(1, 371) = 7.59, *P* = .006). Simple-effects tests showed that participants in the growth (vs. fixed) mindset condition showed lower fixedness both in beauty (*M*_growth/beauty_ = 4.04, SD = 1.36 vs. *M*_fixed/beauty_ = 4.50, SD = 1.19, *P* = .02) and morality (*M*_growth/morality_ = 3.28, SD = 1.51 vs. *M*_fixed/morality_ = 4.53, SD = 1.43, *P* < .001).

**Study 9.** A 2 (Beauty mindset: growth vs. fixed) × 2 (Domain: beauty vs. intelligence) ANOVA on the perceived fixedness of beauty (vs. intelligence) showed a significant main effect of beauty mindset, with fixed (vs. growth) mindset theorists indicating higher fixedness of the attribute (*M*_fixed_ = 4.18, SD = 1.80 vs. *M*_growth_ = 2.66, SD = 1.38, *F*(1, 389) = 90.70, *P* < .001), a non-significant main effect of domain (*P* > .27), and a significant interaction effect of mindset and domain (*F*(1, 389) = 11.67, *P* < .001). Simple-effects tests show that growth-mindset (vs. fixed-mindset) theorists indicated lower fixedness in both beauty (*M*_growth/beauty_ = 3.02, SD = 1.38 vs. *M*_fixed/beauty_ = 3.99, SD = 1.74, *F*(1, 389) = 83.08, *P* < .001) and intelligence (*M*_growth/intelligence_ = 2.30, SD = 1.29 vs. *M*_fixed/intelligence_ = 4.36, SD = 1.86, *F*(1, 389) = 18.79, *P* < .001) domains.

**Study 10**. An independent samples t-test showed that participants in the fixed mindset condition indicated higher fixedness of beauty compared to their counterparts in the growth mindset condition (*M*_fixed_ = 4.47, SD = 1.44 vs. *M*_growth_ = 4.03, SD = 1.40; *t*(322) = -2.81, *P* = .005).

**S5. Donation likelihood in Study 1**

Although donation likelihood is *not* our preregistered outcome variable, it would be worth exploring the effect of beauty mindset on it. A binary logistic regression treating beauty mindset as the independent variable and donation likelihood as a dependent variable revealed a marginally significant main effect on donation likelihood across five countries (*B* = -.24, SE = .13, χ^2^ = 3.69, *P* = .055). Participants in the beauty growth mindset condition (51.7% chose “yes”) were more likely to donate than those in the fixed mindset condition (45.6%).

**S6. Control variables included in Study 1 and results including these control variables**

*Control variables.* We included several control variables, including: In the past two years, how frequently did you donate to charities? (0 times/once/ 2 to 4 times/ 5 to 7 times/ 8 to 10 times); Approximately, how much money (in USD) did you donate to charities?; How frequently did you donate to charities for children?; Approximately, how much money (in USD) did you donate to charities for children?; How frequently did you donate to charities which support education? (0 times/once/ 2 to 4 times/ 5 to 7 times/ 8 to 10 times); Approximately, how much money (in USD) did you donate to charities which support education? To what extent do you like charities which support children? To what extent do you like charities which support education? (1 = not at all, 7 = very much).

*Donation amount****.*** Our key preregistered outcome variable is monetary donation amount. We conducted an ANCOVA with beauty mindset (growth mindset vs. fixed mindset) as the independent variable, donation amount as the dependent variable, and all the control variables described above as covariates. Results showed a significant effect of beauty mindset on donation amount (*M*_growth_ = .20, SD = .22 vs. *M*_fixed_ = .17, SD = .21; *F*(1, 969) = 4.61, *P* = .032, *η^2^* = .01). Liking of charities which support education had a significant effect on donation amount (*F*(1, 969) = 9.20, *P* = .002, *η^2^* = .01). Other covariates had no significant effect on donation amount (*P*s > .05).

**S7. Pretest for the charity options in Study 2**

In a separate pretest, 200 participants from Amazon Mturk were shown information about the two charities (as described in the main study) and responded to a question. “Which charity do you think would empower the orphans more?” (1 = charity A, 7 = charity B). A one-sample t-test (compared to the midpoint 4) showed that charity A was perceived to be more empowering than charity B (*M* = 2.20, *t*(199) = -12.76, *P* < .001).

**S8. Control variables included in Study 2 and results including these control variables**

*Control variables.* We included several control variables. First, participants’ ratings of their self-attractiveness might affect how mindsets may influence them (Hong et al., 1999). We captured respondents’ perception of their own beauty by a measure adapted from the literature on mindsets of intelligence (Hong et al., 1999). Specifically, we showed participants pairs of statements in which one statement illustrated a high level of affirmation for their beauty and was pitted against another statement illustrating a low level of such affirmation. These items included, “I usually think I am attractive” versus “I wonder if I am attractive”, “I am pretty confident that I am attractive” versus “I am not very confident that I am attractive”, and “I am sure that I look attractive” versus “I am not sure that I look attractive”. Participants indicated the extent to which their chosen statement was true for them (1 = a little true, 5 = extremely true; α = .90). Second, participants’ perceived importance of being beautiful was considered. To the extent that beauty is considered important to participants, their belief in its malleability may play a role in their behavior. The perceived importance of being beautiful was measured with six items adapted from the literature (Crocker et al., 2003): “Being good-looking can significantly change my future”, “Being good looking is very important to me”, “Attractiveness is important for my success in life”, “When I think I look attractive, I feel good about myself”, “How I feel about myself is influenced by how attractive I think I am”, and “My sense of self-worth suffers whenever I think I do not look good” (1 = strongly disagree, 7 = strongly agree; α = .80). Third, we measured empathy as an individual difference with four items: (1) Other people’s misfortunes do not disturb me a great deal; (2) I am not really interested in how other people feel; (3) I have tender, concerned feelings for people less fortunate than me; (4) I enjoy making other people feel better (1 = strongly disagree, 7 = strongly agree; α = .60). Finally, we included the Positive and Negative Affect Schedule (PANAS) scale (Watson et al., 1988) to measure mood.

*Donation amount****.*** An ANCOVA with participants’ perception of their own beauty, perceived importance of beauty, empathy, and positive and negative mood as covariates showed that the effect of beauty mindset on donation to the empowering charity remained significant (*M*_growth_ = .70, SD = .25 cents vs. *M*_fixed_ = .61, SD = .31, *F*(1, 204) = 5.27, *P* = .023, *η^2^* = .025). All covariates had no significant effect on donation to the empowering charity (*P*s > .5).

**S9. Detailed experimental procedure in Study 3**

**Method**

Four hundred and twenty-nine Mexicans who are fluent in English on Prolific (46.2% females, *M*_age_ = 26.57, SD = 7.69) participated in the study. Participants were told that they will be asked some information about the COVID-19 vaccination. First, they indicated whether they “have been vaccinated for COVID-19?” (yes/no). Then, they indicated to which extent they agree or disagree with the three statements about beauty, which is our beauty mindset measure (α = .83).

We included several COVID-19 variables that may influence vaccination behavior, including: “Have you ever got tested positive for COVID-19?”; “Has anyone you know got COVID-19?”, and “Is there any incentive program for the COVID-19 vaccination in your area/state?” (1 = yes, 0 = no). Further, we measured perceived vulnerability (Chen et al., 2021) with three items: “How likely do you think you are to contract COVID-19 over the next month?” (1= very unlikely; 7 = very likely); “In the last week, how often have you worried about catching COVID-19?” (1 = never, 7 = all the time)”; “What do you think your chances of getting COVID-19 over the next month are compared with others outside your family?” (1 = no chance, 7 = certain); and response efficacy (Chen et al., 2021) with two items: “Vaccination is a very effective way to protect people against COVID-19”, “Vaccination greatly reduces the risk of catching COVID-19” (1 = strongly disagree, 7 = strongly agree). Finally, we included measures for perception of one’s own beauty and perceived importance of beauty as in Study 2.

**Results**

We conducted a logistic regression with beauty mindset and all the other variables as independent variables, and whether or not participants have been vaccinated as the dependent variable (yes = 1, no = 0) (SI Table S1). Results revealed a significant negative effect of beauty mindset on vaccination status, indicating that the higher the tendency of participants holding a beauty growth mindset, the more likely participants have been vaccinated (*Β* = -.16, *SE* = .08, Wald χ^2^ = 4.55, *P* = .033). Infection status of participants (i.e., whether they have got tested positive for COVID-19), availability of incentive program for vaccination, perceived vulnerability, response efficacy, and perceived importance of beauty had no significant effect on participants’ vaccination status (Infection status: *Β* = -.16, *SE* = .25, Wald χ^2^ = .40, *P* = .526; availability of incentive program for vaccination: *Β* = -.14, *SE* = .27, Wald χ^2^ = .28, *P* = .595; perceived vulnerability: *Β* = -.08, *SE* = .08, Wald χ^2^ = 1.18, *P* = .278; response efficacy: *Β* = .13, *SE* = .09, Wald χ^2^ = 2.45, *P* = .117; perceived importance of beauty: *Β* = .04, *SE* = .09, Wald χ^2^ = .17, *P* = .684). Further, perception of one’s own beauty had a significant positive effect on vaccination status (*Β* = .09, *SE* = .04, Wald χ^2^ = 7.55, *P* = .006), whereas infection status of participants’ social circle (i.e., whether anyone they know has got COVID-19) had a marginally significant negative effect on vaccination status (*Β* = -.89, *SE* = .47, Wald χ^2^ = 3.53, *P* = .060).

**S10. Intervention message (herd protection vs. self protection) used in Study 4 (France), Study 5 (US), and Study 6 (Australia)**

**Study 4 (France):**

*Self-protection condition:*

**Get vaccinated to protect yourself**

COVID-19 vaccines are effective at protecting you from getting sick, especially severe illness and death. Recent data suggests that COVID-19 vaccines can reduce the risk of hospitalization and death by more than 90 percent against the original virus and the highly transmissible Delta variant. The vaccines are designed to trigger the immune system to make antibodies to the spike protein of the virus. This means if you were to ever get the COVID-19 virus, your body is better prepared to fight the illness. Current evidence shows that people who have received a COVID-19 vaccine have a much lower chance of developing more serious symptoms from COVID-19. This is compared to those who did not get the vaccine. Thus, get vaccinated as soon as a vaccine becomes available to protect yourself.

*Herd protection condition:*

**Get vaccinated to achieve herd immunity**

The coronavirus that causes COVID-19 pandemic has spread quickly across communities. Stopping it requires a significant percentage of people to be immune, and thus it is important to get as many people vaccinated as possible. When most of a population is immune to a virus, this provides indirect protection – or population immunity, also called herd immunity or herd protection– to those who are not immune to the virus. For example, if 80% of a population is immune to a virus, four out of every five people who encounter someone with the disease won’t get sick, and won’t spread the disease any further. According to the experts, with the Delta variant, we would need at least 75% of France’s population to be immune to keep the rate of infection down without restrictions on activities. The fastest way to achieve herd immunity is for each of us to do our part to reduce the spread of the virus; that is, to get vaccinated as soon as a vaccine becomes available to us.

**Study 5 (US):**

*Self-protection condition:*

**Get vaccinated to protect yourself**

COVID-19 vaccines are effective at protecting you from getting sick, especially severe illness and death. Based on what we know about COVID-19 vaccines, people who have been fully vaccinated can start resume activities that they did prior to the pandemic. Specifically, if you’ve been fully vaccinated, you can resume activities without wearing a mask or staying 6 feet apart, except where required by federal, state, local, tribal, or territorial laws, rules, and regulations, including local business and workplace guidance. If you travel in the United States, you do not need to get tested before or after travel or self-quarantine after travel. If you’ve been around someone who has COVID-19, you do not need to stay away from others or get tested unless you have symptoms, or unless you live or work in a correctional or detention facility or a homeless shelter. Thus, get vaccinated as soon as a vaccine becomes available to protect yourself.

*Herd protection condition:*

**Get vaccinated to achieve herd immunity**

The coronavirus that causes COVID-19 pandemic has spread quickly across communities. Stopping it requires a significant percentage of people to be immune, and thus it is important to get as many people vaccinated as possible. When most of a population is immune to a virus, this provides indirect protection – or population immunity, also called herd immunity or herd protection– to those who are not immune to the virus. For example, if 80% of a population is immune to a virus, four out of every five people who encounter someone with the disease won’t get sick, and won’t spread the disease any further. What we know about coronavirus so far suggests that we would need at least 70% of the population to be immune to keep the rate of infection down without restrictions on activities. The fastest way to achieve herd immunity is for each of us to do our part to reduce the spread of the virus; that is, to get vaccinated as soon as a vaccine becomes available to us.

**Study 6 (Australia):**

*Self-protection condition:*

**Get vaccinated to protect yourself**

 COVID-19 vaccines are effective at protecting you from getting sick, especially severe illness and death. Recent data suggests that COVID-19 vaccines can reduce the risk of hospitalization and death by more than 90 percent against the original virus and the highly transmissible Delta variant. The vaccines are designed to trigger our immune system to make antibodies to the spike protein of the virus. This means if you were to ever get the COVID-19 virus, your body is better prepared to fight the illness. Current evidence shows that people who have received a COVID-19 vaccine have a much lower chance of developing more serious symptoms from COVID-19. This is compared to those who did not get the vaccine. According to the experts, the possibility of quarantine-free travel and lighter COVID-19 restrictions being available to vaccinated individuals is an absolutely reasonable thing to expect in Australia in the future. Thus, get vaccinated as soon as a vaccine becomes available to protect yourself.

*Herd protection condition:*

**Get vaccinated to achieve herd immunity**

The coronavirus that causes COVID-19 pandemic has spread quickly across communities. Stopping it requires a significant percentage of people to be immune, and thus it is important to get as many people vaccinated as possible. When most of a population is immune to a virus, this provides indirect protection – or population immunity, also called herd immunity or herd protection– to those who are not immune to the virus. For example, if 80% of a population is immune to a virus, four out of every five people who encounter someone with the disease won’t get sick, and won’t spread the disease any further. According to the experts, with the Delta variant, we would need at least 70% of Australia’s population to be immune to keep the rate of infection down without restrictions on activities. The fastest way to achieve herd immunity is for each of us to do our part to reduce the spread of the virus; that is, to get vaccinated as soon as a vaccine becomes available to us.

**S11. Study 4 detailed results (without including covariates)**

*Likelihood to get vaccinated.* Only 56 participants had not been vaccinated; this sample was too small for us to analyze the results for likelihood to get vaccinated. Thus, in this study we only analyzed the data for the likelihood to recommend the vaccine to others, as reported below.

*Likelihood to recommend the vaccine to others*. We conducted a regression analysis with beauty mindset (mean-centered), intervention message (herd protection vs. self protection) and their interaction term as the independent variables, and likelihood to recommend the vaccine as the dependent variable. Results showed that the main effect of beauty mindset was not significant (*P* = .47), and the main effect of intervention message was not significant (*P* = .07). Importantly, the interaction effect of beauty mindset and intervention message was significant (*B* = -.42, *SE* = .14, *t* = -3.01, *P* = .003). Specifically, a moderation analysis (Hayes’ Process, model 1) showed that in herd protection conditions, beauty growth-mindset was associated with greater likelihood to recommend the vaccine (*M*_growth/herd immunity_ = 6.11 vs. *M*_fixed/herd immunity_ = 5.29, *B* = -.35, *SE* = .10, *t* = -3.51, *P* = .0005). The effect was not significant in the self protection conditions (*M*_growth/protect oneself_ = 5.95 vs. *M*_fixed/protect oneself_ = 6.12, *B* = .07, SE = .10, *t* =.73, *P* = .47).

**S12. Study 5 detailed results (without including covariates)**

*Likelihood to recommend the vaccine to others.* A 2 (Beauty mindset: growth vs. fixed) × 2 (Intervention message: herd protection vs. self protection) ANOVA on the likelihood to recommend others to get vaccinated showed a significant main effect of beauty mindset, with growth-mindset theorists being more likely to recommend than fixed-mindset theorists (*M*_growth_ = 6.14, SD = 1.38 vs. *M*_fixed_ = 5.81, SD = 1.96; *F*(1, 419) = 4.23, *P* = .04, *η^2^* = .01), a non-significant main effect of intervention message (*P* > .6), and importantly, a significant interaction effect of beauty mindset and intervention message (*F*(1, 419) = 5.07, *P* = .025, *η^2^* = .01). Follow-up simple-effects test showed that when the intervention message was about herd protection, growth-mindset theorists demonstrated higher likelihood to recommend the vaccine to others compared to fixed-mindset theorists (*M*_growth/herd immunity_ = 6.38 , SD = 1.11 vs. *M*_fixed/herd immunity_ = 5.67, SD = 2.03; *P* = .003), whereas when the intervention message was about self protection, this effect disappeared (*M*_growth/protect oneself_ = 5.93, SD = 1.56 vs. *M*_fixed/protect oneself_ = 5.96, SD = 1.87; *P* = .89).

*Likelihood to get vaccinated.* We further examined the likelihood to get vaccinated among those who have not been vaccinated yet. We note that the sample size for this group was small (*N* = 107; 45.9% females, *M*_age_ = 32.69, SD = 10.89), however we proceeded with the analysis to observe the trends. We conducted a 2 (Beauty mindset: growth vs. fixed) × 2 (Intervention message: herd protection vs. self protection) ANOVA on likelihood to get the vaccine. Results showed a significant main effect of beauty mindset (*M*_growth_ = 4.94, SD = 1.87 vs. *M*_fixed_ = 4.00, SD = 2.42; *F*(1, 105) = 5.67, *P* = .019, *η^2^* = .05), a non-significant main effect of intervention message (*P* > .6), and a marginally significant interaction effect of beauty mindset and intervention message (*F*(1, 105) = 2.91, *P* = .091, *η^2^* = .027). Simple-effects tests showed that when the message was about herd protection, growth-mindset (vs. fixed-mindset) theorists exhibited higher likelihood to get the vaccine (*M*_growth/herd immunity_ = 5.32, SD = 1.39 vs. *M*_fixed/herd immunity_ = 3.57, SD = 2.44; *P* = .009), whereas this difference was not significant when the message was about self protection (*M*_growth/protect oneself_ = 4.77, SD = 2.05 vs. *M*_fixed/protect oneself_ = 4.48, SD = 2.35; *P* = .60).

**S13. Study 6 and follow-up study detailed results (without including covariates):**

**Study 6 results:**

*Likelihood to get vaccinated.* We conducted a multiple regression with likelihood to get vaccinated as the dependent variable, and beauty mindset (mean-centered), intervention message (herd protection vs. self-protection), and their interaction term as the independent variables. Results revealed a non-significant main effect of beauty mindset (*Β* = .08, *SE* = .10, *t* = .77, *P* = .44) and a non-significant main effect of intervention message (*Β* = -.004, *SE* = .19, *t* = -.02, *P* = .98). Importantly, the interaction of beauty mindset and intervention message was significant (*Β* = -.44, *SE* = .15, *t* = -2.97, *P* = .003). A moderation analysis (PROCESS model 1 with 5000 samples; Hayes, 2013) showed that when the intervention message was about herd protection, growth-mindset (vs. fixed-mindset) theorists indicated higher likelihood to get vaccinated (*M*_fixed_ = 5.26 vs. *M*_growth_ = 6.21, *Β* = -.36, *SE* = .11, *t* = -3.40, *P* = .0008), whereas the effect was not significant when the intervention message was about self-protection (*M*_fixed_ = 5.87 vs. *M*_growth_ = 5.66, *Β* = .08, *SE* = .10, *t* = .77, *P* = .44).

*Likelihood to recommend the vaccines to others.* Similarly, we ran a multiple regression with likelihood to recommend the vaccines to others as the dependent variable, and beauty mindset (mean-centered), intervention message (herd protection vs. self-protection), and their interaction term as the independent variables. Results revealed a non-significant main effect of beauty mindset (*Β* = .02, *SE* = .08, *t* = .21, *P* = .84), a non-significant main effect of intervention message (*Β* = -.28, *SE* = .16, *t* = -1.80, *P* = .07), but a significant interaction of beauty mindset and intervention message (*Β* = -.26, *SE* = .12, *t* = -2.20, *P* = .03). A moderation analysis (PROCESS model 1 with 5000 samples; Hayes, 2013) showed that when the intervention message is about herd protection, growth-mindset (vs. fixed-mindset) theorists are more likely to recommend the vaccines to others (*M*_fixed/herd immunity_ = 5.49 vs. *M*_growth/herd immunity_ = 6.13, *Β* = -.24, *SE* = .08, *t* = -2.92, *P* = .004), whereas the difference was not significant when the intervention message was about self-protection (*M*_fixed/protect oneself_ = 6.13 vs. *M*_growth/protect oneself_ = 6.08, *Β* = .02, *SE* = .08, *t* = .21, *P* = .84).

**Follow-up Study to Study 6 (Post-intervention vaccination behavior):**

One month after exposure to our intervention message, we followed up with participants who were not vaccinated at the time of the study. Participants indicated whether they have been vaccinated yet. We conducted a logistic regression with beauty mindset (mean-centered), intervention message (herd protection vs. self-protection), and their interaction term as the independent variables, and vaccination status (1 = yes, 0 = no) as the dependent variable. Results showed that the main effect of beauty mindset was significant (*B* = -.43, SE = .18, Wald χ^2^ = 5.99, *P* = .01), the effect of intervention message (herd protection vs. self-protection) was not significant (*P* = .47), and importantly, the interaction effect of beauty mindset and intervention message was significant (*B* = .65, SE = .26, Wald χ^2^ = 6.45, *P* = .01). Further moderation analysis (Hayes’ Process, model 1) showed that in herd protection conditions, growth-mindset increased vaccination rate (B = -.43, SE = .18, *P* = .01). In the self protection conditions, the effect of beauty mindset on vaccination rate was not significant (*P* = .24).

**S14. Study 4 results including control variables**

*Control variables.* We included several COVID-19 variables that may influence vaccination behavior, including infection status of participants (“Have you ever got tested positive for COVID-19?”), infection status of participants’ social circle (“Has anyone you know got COVID-19?”), and availability of incentive program for vaccination (“Is there any incentive program for the COVID-19 vaccination in your area/state?” (1 = yes, 0 = no). Further, we included measures for perception of one’s own beauty and perceived importance of beauty as in Study 2 and Study 3.

*Likelihood to recommend the vaccine to others.* We conducted a regression analysis with beauty mindset (mean-centered) and intervention message (herd protection vs. self protection), and their interaction term as the independent variables, likelihood to recommend the vaccine as the dependent variable, and the control variables as covariates. Results showed that the main effect of beauty mindset was not significant (*p* = .75), and the main effect of intervention message was not significant (*p* = .11). Importantly, the interaction effect of beauty mindset and intervention message was significant (*B* = -.36, *SE* = .14, *t* = -2.67, *P* = .008). Perception of own attractiveness (*B* = -.07, *SE* = .03, *t* = -2.30, *P* = .02), perceived importance of beauty (*B* = .34, *SE* = .07, *t* = 4.98, *P* < .001), and infection status of participants’ social circle (*B* = -.56, *SE* = .21, *t* = -2.70, *P* = .007) had a significant effect on likelihood to recommend the vaccine. Other control variables had no significant effect (*P*s > .05).

A moderation analysis (Hayes’ Process, model 1) showed that in herd protection conditions, beauty growth mindset was associated with greater likelihood to recommend the vaccine (*M*_growth/herd immunity_ = 6.11 vs. *M*_fixed/herd immunity_ = 5.34, *B* = -.33, *SE* = .10, *t* = -3.44, *P* = .0007). The effect was not significant in the self protection conditions (*M*_growth/protect oneself_ = 5.97 vs. *M*_fixed/protect oneself_ = 6.05, *B* = .03, *SE* = .10, *t* = .33, *P* = .74).

**S15. Study 5 results including control variables**

*Control variables.* We included the same control variables as in Study 4, including infection status of participants, infection status of participants’ social circle, availability of incentive program for vaccination, perception of one’s own beauty and perceived importance of beauty.

*Likelihood to recommend the vaccine to others.* An ANCOVA with the control variables mentioned above as covariates showed a marginally significant main effect of beauty mindset (*F*(1, 410) = 3.44, *P* = .064, *η^2^* = .01), a non-significant main effect of intervention message (*F*(1, 410) = .08, *P* = .782, *η^2^* < .001), and a significant interaction effect of beauty mindset and intervention message (*F*(1, 410) = 4.00, *P* = .046, *η^2^* = .01). Specifically, growth-mindset (vs. fixed-mindset) theorists showed higher intention to recommend the vaccine to others when the intervention message is about herd protection (*M*_growth/herd protection_ = 6.38 , *SD* = 1.11 vs. *M*_fixed/herd protection_ = 5.72 , *SD* = 2.00, *F*(1, 410) = 7.37, *P* = .007*, η^2^* = .02 ) whereas the effect was non-significant when the intervention message was about self protection (*P* = .92). Perception of one’s own beauty (*F*(1, 410) = 10.98, *P* = .001, *η^2^* = .03), infection status of participants (*F*(1, 410) = 3.89, *P* = .049, *η^2^* = .01), and infection status of participants’ social circle (*F*(1, 410) = 13.21, *P* < .001, *η^2^* = .03) had significant effects on likelihood to recommend the vaccine to others. Other control variables had no significant effect (*P*s > .05).

*Likelihood to get vaccinated.* Again we note that the sample size for this group was small (*N* = 107). We conducted a 2 (Beauty mindset: fixed vs. growth) × 2 (Intervention message: herd protection vs. self protection) ANCOVA on likelihood to get the vaccine, including the control variables as covariates. Results showed a significant main effect of beauty mindset (*F*(1, 98) = 7.21, *P* = .009, *η^2^* = .07), a non-significant main effect of intervention message (*F*(1, 98) = .07, *P* = .80, *η^2^* = .001), and a non-significant interaction effect of beauty mindset and intervention message (*F*(1, 98) = 2.63, *P =* .11, *η^2^* = .03). However, simple-effects tests showed that when the intervention message is about herd protection, growth-mindset (vs. fixed-mindset) theorists exhibited higher likelihood to get the vaccine (*M*_growth/herd protection_ = 5.32, SD = 1.39 vs. *M*_fixed/herd protection_ = 3.59, SD = 2.49, *F*(1, 98) = 7.99, *P* = .006, *η^2^* = .08), whereas this difference was not significant when the intervention message was about self protection (*M*_growth/self protection_ = 4.74, SD = 2.07 vs. *M*_fixed/self protection_ = 4.48, SD = 2.35, *F*(1, 98) = .69, *P* = .41, *η^2^* = .007). Availability of incentive program for vaccination (*F*(1, 98) = 4.14, *P* = .045, *η^2^* = .04) and perceived importance of beauty (*F*(1, 98) = 3.94, *P* = .050, *η^2^* = .04) had significant effects on likelihood to get vaccinated. Other control variables had no significant effect (*P*s > .05).

**S16. Study 6 and follow-up study results including control variables**

**Study 6 results:**

*Control variables.* We included the same control variables as in Studies 4 and 5, including infection status of participants, infection status of participants’ social circle, availability of incentive program for vaccination, perception of one’s own beauty and perceived importance of beauty.

*Likelihood to get vaccinated.* We conducted a multiple regression with likelihood to get vaccinated as the dependent variable, and beauty mindset (mean-centered), intervention message (herd protection vs. self protection), their interaction term as the independent variables, and the control variables described above as covariates. Results revealed a non-significant main effect of beauty mindset (*Β* = .07, *SE* = .10, *t* = .67, *P* = .50) and a non-significant main effect of intervention message (*Β* = .01, *SE* = .19, *t* = .07, *P* = .95). Importantly, the interaction of beauty mindset and intervention message was significant (*Β* = -.45, *SE* = .15, *t* = -3.07 *P* = .002). Perceived importance of beauty had a significant effect on vaccination likelihood (*B* = .27, *SE* = .08, *t* = 3.21, *P* = .001). Other covariates had no significant effect (*P*s > .2).

A moderation analysis (PROCESS model 1 with 5000 samples; Hayes, 2013) including control variables showed that when the intervention message was about herd protection, growth-mindset (vs. fixed-mindset) theorists indicated higher likelihood to get vaccinated (*M*_fixed_ = 5.23 vs. *M*_growth_ = 6.24, *Β* = -.38, *SE* = .11, *t* = -3.59, *P* = .0004), whereas the effect was not significant when the intervention message was about self protection (*M*_fixed_ = 5.85 vs. *M*_growth_ = 5.67, *Β* = .07, *SE* = .10, *t* = .67, *P* = .50).

*Likelihood to recommend the vaccines to others.* Similarly, we ran a multiple regression with likelihood to recommend the vaccines to others as the dependent variable, and beauty mindset (mean-centered), intervention message (herd protection vs. self protection), their interaction term as the independent variables, and the control variables as covariates. Results revealed a non-significant main effect of beauty mindset (*Β* = .04, *SE* = .08, *t* = .50, *P* = .62), a non-significant main effect of intervention message (*Β* = -.26, *SE* = .15, *t* = -1.73, *P* = .09), but a significant interaction of beauty mindset and intervention message (*Β* = -.26, *SE* = .12, *t* = -2.28, *P* = .02). The control variables had no significant effect (*P*s > .05).

A moderation analysis (PROCESS model 1 with 5000 samples; Hayes, 2013) including the control variables showed that when the intervention message was about herd protection, growth-mindset (vs. fixed-mindset) theorists are more likely to recommend the vaccine to others (*M*_fixed_ = 5.52 vs. *M*_growth_ = 6.11, *Β* = -.22, *SE* = .08, *t* = -2.69, *P* = .008), whereas this difference was not significant when the intervention message was about self protection (*M*_fixed_ = 6.16 vs. *M*_growth_ = 6.04, *Β* = .04, *SE* = .08, *t* = .50, *P* = .62).

**Follow-up Study to Study 6 (Post-intervention vaccination behavior)**

After one month, we followed up with participants who were not vaccinated at the time of the study. Participants indicated whether they have been vaccinated yet. We conducted a logistic regression with beauty mindset (mean-centered), intervention message (herd protection vs. self protection), and their interaction term as the independent variables, vaccination status (1 = yes, 0 = no) as the dependent variable, and the control variables measured in Study 6 as covariates. Results showed that the main effect of beauty mindset was not significant (*P* = .17), the effect of intervention message was not significant (*P* = .42), but importantly, the interaction effect of beauty mindset and intervention message was significant (*B* = -.66, *SE* = .26, Wald χ^2^ = 6.61, *P* = .01). Among the covariates, only infection status of participants’ social circle had a significant effect on vaccination status (*B* = -.80, *SE* = .36, Wald χ^2^ = 5.01, *P* = .03). Further moderation analysis (Hayes’ Process, model 1) including the control variables showed that in herd protection conditions, growth-mindset increased vaccination rate (*B* = -.40, *SE* = .18, *P* = .02) compared to fixed-mindset. In the self protection conditions, the effect of beauty mindset on vaccination rate was not significant (*P* = .17).

**S17. Study 7 – Control variables and results including control variables**

*Control variables*. We measured the same control variables as in study 2, namely participants’ perception of their own beauty, perceived importance of beauty, and mood. As mentioned in the main text, in this study we also measured long-term orientation with three items: “Right now, I am planning for the long term”. “Right now, I am thinking about success in the future”, and “Right now, I don’t mind giving up today’s immediate outcomes for success in the future” (1 = strongly disagree, 7 = strongly agree; α **=** .84; adapted from the literature [Bearden et al., 2006; Kopalle et al., 2010]). Further, to control for participants’ actual voter registration, we included a question: “Many people are not registered to vote because they are too busy or move around often. Would official state records show that you: 1 = are registered to vote, 0 = are not now registered to vote”.

*Voting.* An ANCOVA analysis with the control variables (perception of own beauty, perceived importance of beauty, positive and negative mood, long-term orientation, and actual voter registration) showed that the effect of beauty mindset (growth vs. fixed vs. control) on voting intentions remained significant (*F*(2, 364) = 3.84, *P* = .022, *η^2^* = .02). Perceived importance of beauty (*F*(1, 364) = 3.91, *P* = .049, *η^2^* = .01), long-term orientation (*F*(1, 364) = 4.75, *P* = .03, *η^2^* = .01), and actual voter registration (*F*(1, 364) = 124.98, *P* < .001, *η^2^* = .26) had significant effects on voting likelihood. Other control variables had no significant effect (*P*s > .05).

**S18. Study 8 – Morality mindset stimuli**


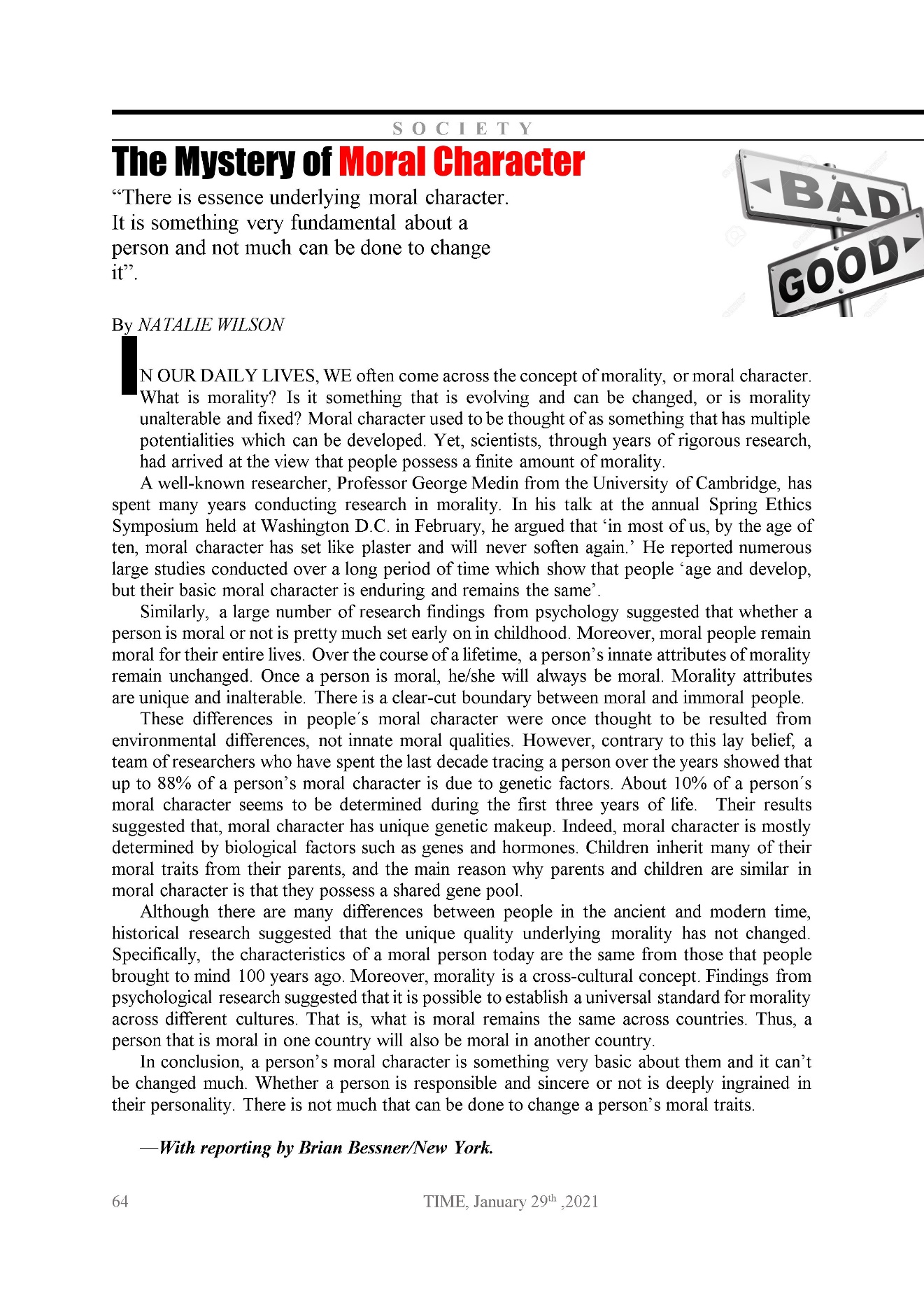


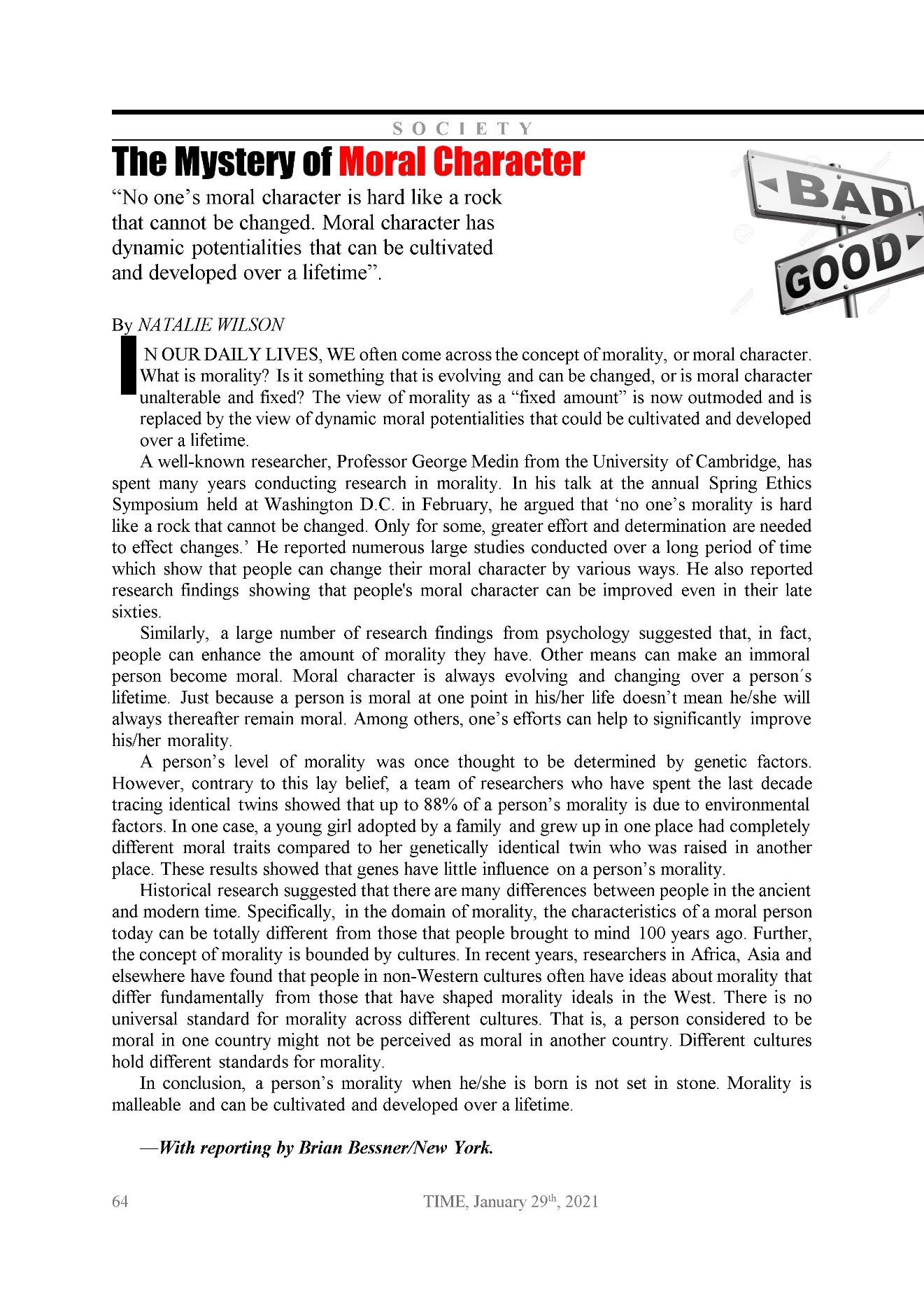


**S19. Study 9 – Intelligence mindset stimuli**
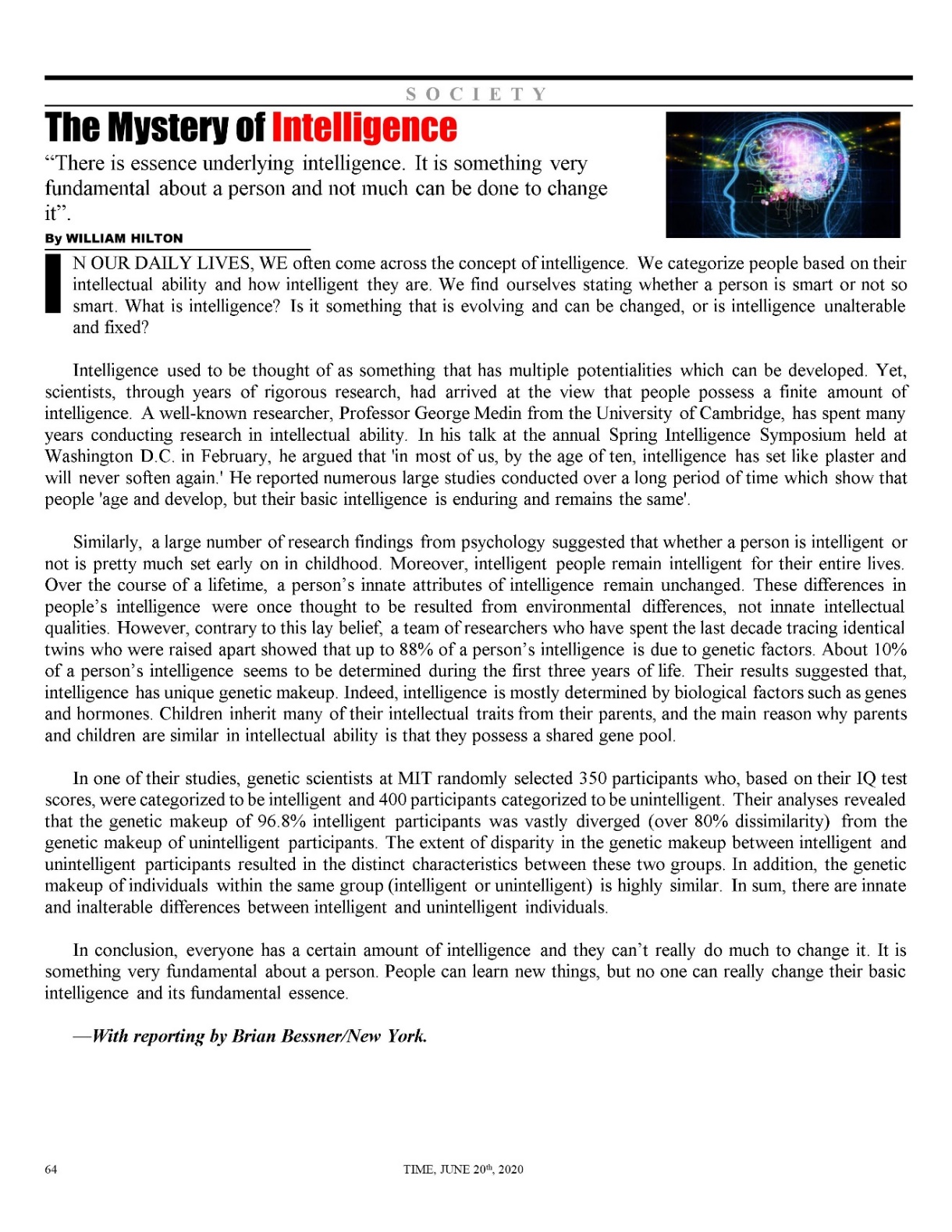


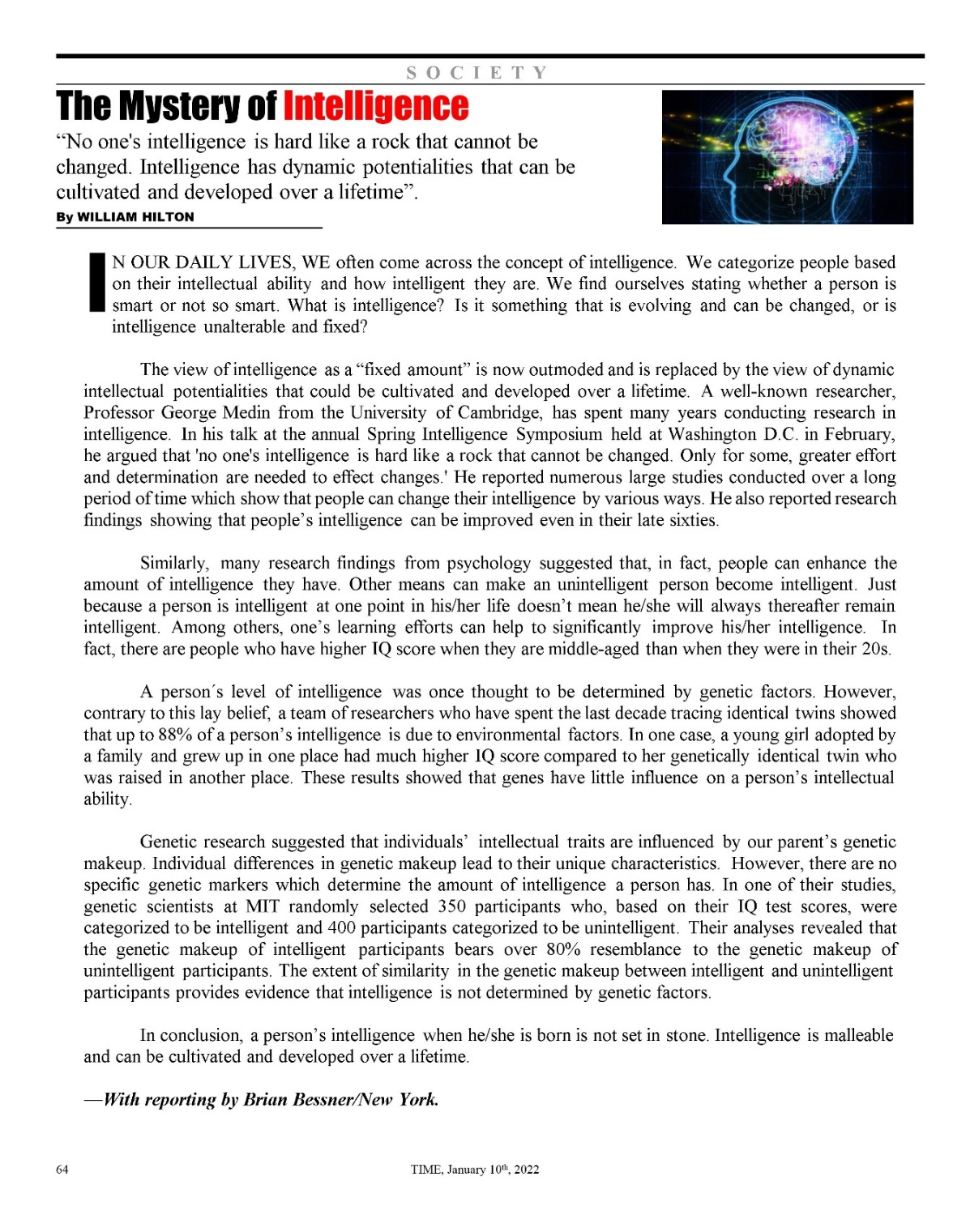


**S20. Detailed information shown to participants in Study 8 and Study 9.**

“As researchers, we work with organizations who are trying to address different social challenges. This year, we are working with the International Domestic Violence Action Center, whose mission is to end domestic violence and other forms of abuse. At the moment, the Center is having an online petition to raise awareness about domestic violence and to gather support to shelters that provide housing for victims of domestic violence”.

**S21. Study 8 results including control variables**

We measured similar control variables as in previous studies, including perception of one’s own beauty (vs. morality), perceived importance of beauty (vs. morality), empathy, and mood.

*Willingness to sign the petition.* An ANCOVA with the control variables (i.e., perception of one’s own beauty (vs. morality), perceived importance of beauty (vs. morality), empathy, and positive and negative mood) as covariates revealed that the interaction effect of beauty mindset and domain became marginally significant (*F*(1, 366) = 2.76, *P* = .097, *η^2^* = .01). The main effects of beauty mindset and domain were not significant (*P*s > .2). Follow-up simple-effects tests revealed that in the beauty domain, participants in the growth (vs. fixed) mindset condition were more willing to sign the petition, although the effect was only marginally significant (*M*_growth/beauty_ = 5.98, SD = 1.26 vs. *M*_fixed/beauty_ = 5.51, SD = 1.61; *F*(1, 366) = 2.92, *P* = .088, *η^2^* = .01). In contrast, this difference was not significant in morality (*M*_growth/morality_ = 5.65, SD = 1.79 vs. *M*_fixed/morality_ = 5.83, SD = 1.35; *F*(1, 366) = .44, *P* = .509, *η^2^* = .001). Among the covariates, only empathy had a significant effect on willingness to sign the petition (*F*(1, 366) = 26.98, *P* < .001, *η^2^* = .07). Other control variables had no significant effect (*P*s > .05).

**SI 22. Study 10 results including control variables.**

An ANCOVA analysis with the control variables (i.e., perception of one’s own beauty, perceived importance of beauty, and mood) as covariates showed that the effect of beauty mindset on support for the stigmatized identity remained significant (*M*_growth_ = 3.56, SD = 1.73 vs. *M*_fixed_ = 2.99, SD = 1.60, *F*(1, 318) = 9.90, *P* = .002, *η^2^* = .03). Among the covariates, only positive mood had a significant effect on support for the stigmatized identity (*F*(1, 318) = 4.61, *P* = .03, *η^2^* = .01). Other control variables had no significant effect (*P*s > .05).

**Table S1**. Logistic regression results in Study 3

| **Variables** | ***B*** | ***SE*** | **Wald χ^2^** | ***P* value** |
| --- | --- | --- | --- | --- |
| Beauty mindset | -.162 | .076 | 4.550 | .033 |
| Have you ever got tested positive for covid-19? | -.158 | .249 | .401 | .526 |
| Has anyone you know got covid-19? | -.887 | .472 | 3.530 | .060 |
| Is there any incentive program for the covid-19 vaccination in your area/state? | -.142 | .268 | .282 | .595 |
| Perceived vulnerability | -.084 | .077 | 1.178 | .278 |
| Response efficacy | .133 | .085 | 2.453 | .117 |
| Perception of one’s own beauty | .096 | .035 | 7.549 | .006 |
| Perceived importance of beauty | .037 | .090 | .166 | .684 |

**References**

1. Faust, N. T., Hong, Y.-y., Gains, N. & Christopoulos, G. I. The effect of implicit theories of human beauty and perceived pressure on cosmetic consumption. *Psychol. Aesthet. Create. Arts.* **18**, 43–58 (2024).

2. Hong, Y., Chiu, C., Dweck, C. S., Lin, D. M. S. & Wan, W. Implicit theories, attributions, and coping: A meaning system approach. *J. Pers. Soc. Psychol*. **77**, 588–599 (1999).

3. Crocker, J., Luhtanen, R. K., Cooper, L. M. & Bouvrette, A. Contingencies of self-worth in college students: Theory and measurement. *J. Pers. Soc. Psychol*. **85**, 894-908 (2003).

4. Watson., D., Clark, L. A. & Tellegen, A. Development and validation of brief measures of positive and negative affect: The PANAS scales. *J. Pers. Soc. Psychol*. **54**, 1063-1070 (1988).

5. Chen, Y. et al. Differences in the protection motivation theory constructs between people with various latent classes of motivation for vaccination and preventive behaviors against covid-19 in Taiwan. *Int. J. Environ. Res. Public Health.* **18**, 7042 (2021).

6. Hayes, A. F. *Introduction to Mediation, Moderation, and Conditional Process Analysis: A Regression Based Approach* (The Guilford Press, 2013).

7. Bearden, W. O., Money, R. B. & Nevins, J. L. A measure of long-term orientation: Development and validation. *J. Acad. Mark. Sci.* **34**, 456–467 (2006).

8. Kopalle, P.K., Lehmann., D.R. & Farley, J.U. Consumer expectations and culture: The effect of belief in karma in India. *J. Consum. Res.* **37**, 251– 263 (2010).
